# Alterations in Intestinal Brush Border Membrane Functionality and Bacterial Populations Following Intra-Amniotic Administration (*Gallus gallus*) of Nicotinamide Riboside and Its Derivatives

**DOI:** 10.3390/nu14153130

**Published:** 2022-07-29

**Authors:** Nikolai Kolba, Amin Zarei, Jacquelyn Cheng, Nikita Agarwal, Younas Dadmohammadi, Leila Khazdooz, Alireza Abbaspourrad, Elad Tako

**Affiliations:** Department of Food Science, Cornell University, Ithaca, NY 14853, USA; nk598@cornell.edu (N.K.); az534@cornell.edu (A.Z.); jyc53@cornell.edu (J.C.); na494@cornell.edu (N.A.); younas@cornell.edu (Y.D.); lk532@cornell.edu (L.K.)

**Keywords:** intra-amniotic administration, brush border membrane, nicotinamide riboside derivatives, microbiome

## Abstract

Nicotinamide riboside (NR) acts as a nicotinamide adenine dinucleotide (NAD+) precursor where NR supplementation has previously been shown to be beneficial. Thus, we synthesized and characterized nicotinamide riboside tributyrate chloride (NRTBCl, water-soluble) and nicotinamide riboside trioleate chloride (NRTOCl, oil-soluble) as two new ester derivatives of nicotinamide riboside chloride (NRCl). NRCl and its derivatives were assessed in vivo, via intra-amniotic administration (*Gallus gallus*), with the following treatment groups: (1) non-injected (control); and injection of (2) deionized H_2_O (control); (3) NRCl (30 mg/mL dose); (4) NRTBCl (30 mg/mL dose); and (5) NRTOCl (30 mg/mL dose). Post-intervention, the effects on physiological markers associated with brush border membrane morphology, intestinal bacterial populations, and duodenal gene expression of key proteins were investigated. Although no significant changes were observed in average body weights, NRTBCl exposure increased average cecum weight. NR treatment significantly increased *Clostridium* and NRCl treatment resulted in increased populations of *Bifidobacterium*, *Lactobacillus*, and *E. coli*. Duodenal gene expression analysis revealed that NRCl, NRTBCl, and NRTOCl treatments upregulated the expression of ZnT1, MUC2, and IL6 compared to the controls, suggesting alterations in brush border membrane functionality. The administration of NRCl and its derivatives appears to trigger increased expression of brush border membrane digestive proteins, with added effects on the composition and function of cecal microbial populations. Additional research is now warranted to further elucidate the effects on inflammatory biomarkers and observe changes in the specific intestinal bacterial populations post introduction of NR and its derivatives.

## 1. Introduction

The duodenal brush border membrane (BBM), the digestive and absorptive surface of the small intestine, is an essential component of the digestive tract where elucidation of gut health can be ascertained by BBM functionality and morphology [1]. A critical factor in intestine health maintenance is its resident microbiota’s composition and function [1]. The intestinal microbiota comprises trillions of microorganisms that live symbiotically with their host. These microbes play vital roles in the digestive and immunological functions of the gastrointestinal tract by preventing colonization of potentially pathogenic organisms regulating the mucosal immune system, and maintaining intestinal homeostasis [2]. Further, this commensal microbial community contributes to the digestion of dietary fibers and minerals [3,4]; and interacts with epithelial cells to maintain an effective gut barrier [5,6]. Prebiotics, foods fortified with micronutrients such as vitamins and minerals, and polyphenolic compounds such as anthocyanins have been shown to exert positive effects on the growth and activity of bacteria that improve host health [7,8,9,10,11]. These health-promoting food ingredients are fermented to short-chain fatty acids (SCFAs) by specific bacteria in the colon [12,13,14,15]. The presence of SCFAs has been associated with increasing populations of bacteria (i.e., *Lactobacillus* and *Bifidobacterium*)*,* acidifying the intestinal luminal pH and subsequently inhibiting the growth of potentially pathogenic bacteria.

Butyrate, a well-studied SCFA, plays a critical role in gastrointestinal tract health, where butyrate is utilized as an energy source for intestinal epithelial cells and can assist in maintaining intestinal epithelium integrity. Moreover, butyrate can protect the host from potential immune and inflammatory diseases associated with the translocation of antigens and pathogens [16]. Increased SCFA production has been associated with increased enterocyte proliferation, demonstrated through increases in villus surface area and enhancements in duodenal BBM functionality [17]. BBM functional capacity dictates the extent of food hydrolysis and micronutrient uptake. Thus, it is essential to examine the interactions between dietary bioactives and the BBM functionality and morphology.

It was previously demonstrated that the *Gallus gallus* is physiologically relevant in vivo models for evaluating the absorption and bioavailability of biofortified foods and bioactive compounds [18,19,20,21,22,23,24]. Specifically, the intra-amniotic administration approach, where the amniotic fluid is naturally and orally consumed by the embryo, allows for an assessment of the effects of the solution administered into the amniotic fluid on the different systems of interest. Hence, and as was previously demonstrated, this in vivo model has been utilized to study the impact of various biofortified foods, prebiotics, amino acids, carbohydrates, food additives, and antioxidants on BBM morphology and functionality, the intestinal bacterial populations, and mineral status [6,16,17,25,26,27,28,29,30].

Nicotinamide riboside (NR) is a bioavailable form of vitamin B_3_ naturally present in the diet and acts as a cellular nicotinamide adenine dinucleotide (NAD+) precursor [31,32]. NAD+ is an essential cofactor and substrate for numerous critical cellular processes, where NAD+ precursors were demonstrated to have protective roles in several disease states [33]. NR is reportedly more effective than other NAD+ precursors, such as niacin and nicotinamide [32]. As NR-enriched foods have not yet been identified and well-characterized [34], supplementation of NR as a prebiotic can be a promising option to boost NAD+ levels [31,32,35]. NR supplementation was previously studied, in vivo and in humans, and was found to have beneficial effects, including the reduction of DNA and mitochondria damage [36] and providing therapeutic benefits for Alzheimer’s disease [37], obesity [38,39], diabetes [39,40], muscle degeneration [35], and aging [41].

Moreover, NR has been shown to improve the ability to fight pathogenic infections by increasing the innate immune response [41,42]. Currently, NR is an FDA-approved nutritional supplement and is commercially available as a chloride salt of NR (NRCl) in capsule form under the brand name Niagen^TM^ [43]. Recently, Gonzalez et al. 2020 studied the intra-amniotic administration of NR on myogenesis, where significant beneficial effects were observed with pectoralis muscle development in vivo [34,44]. However, the impact of NR and its derivatives on intestinal health, including BBM functionality and bacterial populations, have not yet been studied.

**Figure 1:**
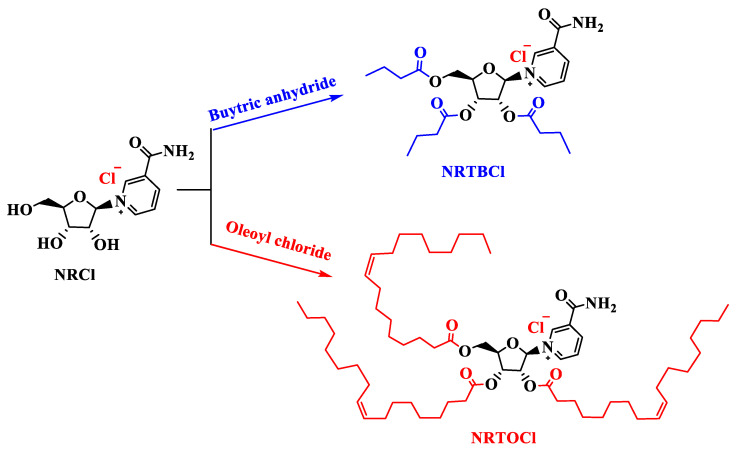
Functionalization of NRCl to NRTBCl and NRTOCl.

In this work, for the first time, we studied the functionalization of currently commercially available NRCl to nicotinamide riboside tributyrate chloride (NRTBCl) as a water-soluble derivative and nicotinamide riboside trioleate chloride (NRTOCl) as an oil-soluble derivative (Figure 1). The primary objective of this study was to assess the effects of intra-amniotic administration of NRCl and two new derivatives (NRTBCl and NRTOCl) on BBM functionality via the evaluation of duodenal gene expression of BBM biomarkers, specifically key digestive and absorptive proteins, immune function proteins, and inflammation biomarkers, in vivo. Further, we assessed the effects of NRCl and its new derivatives on cecal bacterial populations. We hypothesized that intra-amniotic administration of NRCl and its derivatives causes favorable alterations in brush border functionality and gut microbiota.

## 2. Materials and Methods 

### 2.1. Animals

For the following study, a commercial hatchery (Moyer’s chicks, Quakertown, PA, USA) provided Cornish cross-fertile broiler chicken eggs (*n* = 50). The eggs were incubated under controlled conditions at the Cornell University Animal Science poultry farm incubator. All animal protocols were approved by the Cornell University Institutional Animal Care and Use Committee (IACUC #2020-0077).

### 2.2. Materials

Nicotinamide riboside chloride (NRCl, beta form) was received as a donation from ChromaDex Company (Los Angeles, CA, USA). Oleoyl chloride, butyric anhydride, and 4-dimethylamino pyridine were purchased from Sigma Aldrich with 89, 98, and 99% purity, respectively. Pyridine was bought from Fluka with 99.9% purity. Silica gel (P60, 40–63 µm, 60 Å) was purchased from SiliCycle (Québec, QC, Canada), and Silica Gel 60 F254 Coated Aluminum-Backed TLC (thin layer chromatography) sheets were obtained from EMD Millipore (Billerica, MA, USA).

#### 2.2.1. Synthesis of Nicotinamide Riboside Tributyrate Chloride (NRTBCl)

Using a round-bottom flask in an ice bath, 300 mg (1.035 mmol) of NRCl, 25 mg of 4-dimethylamino pyridine (0.205 mmol), 1.5 mL of butyric anhydride (9.170 mmol), and 9 mL of acetonitrile (CH_3_CN) were added and stirred for 5 h under nitrogen blanket. A thin-layer chromatography (TLC) test was employed to track the progress of the reaction. Next, the solvent was evaporated by a rotary evaporator under reduced pressure, and the excess amount of butyric anhydride was washed out with *n*-hexane. Finally, the crude product was purified by column chromatography on SiO_2_. The eluent was a mixture of CH_3_OH (35%) and ethyl acetate (EtOAc) (65%). The purified NR-tributyrate chloride was obtained in 71% (367 mg) as a pale-yellow-colored greasy product (λ_max_ in water was 266 nm).

#### 2.2.2. Synthesis of Nicotinamide Riboside Trioleate Chloride (NRTOCl)

Using a round-bottom flask in an ice bath, 200 mg (0.690 mmol) of NRCl, 0.55 mL (6.81 mmol) of pyridine, and 4.75 mL of dimethylformamide (DMF) were added. Then, 2.0 mL (5.38 mmol) of oleoyl chloride was dropwise added, and the reaction mixture was stirred for 3 h under a nitrogen blanket. A TLC test was used to track the progress of the reaction. After 3 h, 5 mL of methanol was added to the reaction mixture to neutralize the extra amount of oleoyl chloride. After that, the solvent was evaporated using a rotary evaporator under reduced pressure. The crude product was extracted in *n*-hexane and purified using column chromatography on SiO_2_. The eluent was a mixture of CH_3_OH (12%) and EtOAc (88%). The purified NRTOCl was obtained in 64.3% (479.2 mg) as a pale-creamy-colored greasy product (λ_max_ in methanol was 267 nm).

### 2.3. Characterization of NRTBCl and NRTOCl

#### 2.3.1. Nuclear Magnetic Resonance (NMR) Spectroscopy

A 500 MHz NMR (Bruker AVANCE) spectrometer was used for ^1^H NMR (500 MHz) and ^13^C NMR (125 MHz) spectra in deuterated chloroform (CDCl_3_). The chemical shifts were expressed in δ (ppm) relative to tetramethylsilane (TMS) as the internal standard and coupling constants (J) were measured in Hz. Spin multiplicities were described as singlet (s), doublet (d), triplet (t), quartet (q), and multiplet (m).

#### 2.3.2. Attenuated Total Reflectance—Fourier-Transform Infrared (ATR-FTIR) Spectroscopy

The ATR-FTIR spectra were recorded on a Shimadzu IRAffinity-1S spectrophotometer in transmittance mode in the range of 400–4000 cm^−1^ wave number.

#### 2.3.3. UV-Vis Spectroscopy

UV-Vis was recorded on a Shimadzu UV-2600 spectrophotometer in the range of 200–800 nm.

#### 2.3.4. Liquid Chromatography-Mass Spectrometry (LC-MS) Analysis

Liquid Chromatograph (Agilent 1100 series) was coupled with a mass spectrometer for LC-MS analysis. Before injection, all samples were passed through a 13 mm nylon syringe filter with a 0.22 μm pore size. Reverse-phase chromatography was used with a Phenomenex Luna Omega (Phenomenex) LC column with the following specifications: 100 × 4.6 mm, 3 µm, polar C18, 100 Å pore size with a flow rate of 0.3 mL min^−1^. LC eluents include MilliQ-water (solvent A) and acetonitrile (solvent B) using gradient elution (solution A: B composition change with time: 0 min: 95:5, 3 min: 95:5, 15 min: 85:15, 17 min: 90:10, and 20 min 95:5). The mass spectrometer (Finnigan LTQ mass spectrometer) was equipped with an electrospray interface (ESI) set in positive electrospray ionization mode for analyzing the NRTOCl and NRTBCl. The optimized parameters were sheath gas flow rate at 20 arbitrary units, spray voltage set at 4.00 kV, capillary temperature at 350 °C, capillary voltage at 41.0 V, and tube lens set at 125.0 V.

#### 2.3.5. Particle Characterization

The particle size distribution, mean particle diameter (average zeta size), and zeta-potential of NRTOCl in 1% ethanol in DI water were measured using a commercial dynamic light-scattering device (Nano-ZS, Malvern Instruments, Worcestershire, UK).

### 2.4. Intra-Amniotic Administration Solution Preparation

After the synthesis and characterization of NRTBCl and NRTOCl, these compounds and NRCl were used for intra-amniotic administration. NRCl and NRTBCl were dissolved in DI H_2_O at 30 mg/mL. NRTOCl was insoluble in water; thus, it was dispersed using 1% ethanol as a cosolvent.

### 2.5. Intra-Amniotic Administration Procedure and Study Design

The intra-amniotic administration procedure was previously described by Tako et al. [25,28,30,45,46,47,48]. On Day 17 of embryonic incubation, eggs with viable embryos were weighed and allocated into treatment groups (*n* = 10) with equal weight distribution. The intra-amniotic injection solution (1 mL) was injected with a 21-gauge needle into the amniotic fluid, recognized by candling. Following injection, the injection sites were sterilized with 70% ethanol and sealed with cellophane tape. Eggs were then placed in hatching baskets, with each treatment equally represented at each incubator location. The treatment groups are as follows: (1) non-injected (control); and injection of (2) DI H_2_O (control); (3) NR (30 mg/mL dose); (4) NRTBCl (30 mg/mL dose); and (5) NRTOCl (30 mg/mL dose).

### 2.6. Tissue Collection

Immediately post-hatch (Day 21), birds were weighed and euthanized with CO_2_ exposure. The duodenum, ceca, and pectoral muscles were immediately collected and frozen in liquid nitrogen. Samples were stored at −80 °C until analysis [49].

### 2.7. Isolation of Total RNA from Chicken Duodenum

Total RNA was extracted from 30 mg of the proximal duodenal tissue using a Qiagen RNeasy Mini Kit (Qiagen Inc., Germantown, MD, USA). Total RNA was eluted in 50 μL of RNase-free water. All steps were carried out under RNase-free conditions. RNA was quantified with a NanoDrop 2000 (ThermoFisher Scientific, Waltham, MA, USA) at A 260/280. RNA was stored at −80 °C until use.

### 2.8. Real-Time Polymerase Chain Reaction

All procedures were conducted as previously described [30,47,50]. Briefly, the primers used in the real-time polymerase chain reactions (RT-PCR) were designed using Real-time Primer Design Tool software (IDT DNA, Coralville, IA, USA) based on 11 gene sequences from the GenBank database (Table 1). cDNA was generated using a C1000 Touch thermocycler (Biorad, Hercules, CA, USA) and a Promega-Improm-II Reverse Transcriptase Kit (Catalog #A1250) 20 μL reverse transcriptase reaction following the manufacturer’s protocols. The concentration of cDNA was determined with a NanoDrop 2000 at A 260/280 with an extinction coefficient of 33 for single-stranded DNA.

RT-PCR procedure was conducted with a Bio-RadCFX96 Touch (Hercules, CA, USA). Ten μL RT-PCR mixtures consisted of cDNA (2 μg), 2X BioRad SSO Advanced Universal SYBR Green Supermix (Catalog #1725274, Hercules, CA, USA), forward and reversed primers, and nuclease-free water (no template control). The no-template control of nuclease-free water was included to eliminate DNA contamination in the PCR mix. Reactions were performed in duplicates and under the following reaction conditions: initial denaturing (95 °C, 30 s), followed by 40 cycles of denaturing (95 °C, 15 s), several annealing temperatures (according to IDT for 30 s), and elongating (60 °C, 30 s). After the cycling process was accomplished, melting curves were determined from 65.0 °C to 95.0 °C with increments of 0.5 °C for 5 s to confirm the amplification of a single product. RT-PCR efficiency values for the eleven genes were Muc2, 1.022; 18s rRNA, 0.934. Gene expression levels were determined from Ct values based on the ‘second derivative maximum’ calculated by the Bio-Rad CFX Maestro Software (Bio-Rad, Hercules, CA, USA). Gene expression was standardized to the expression of 18S.

### 2.9. Cecal Microbial DNA Isolation and Analysis

All procedures were conducted as previously described [18,22,24,30,47]. Briefly, Ceca contents were inserted into a sterile 50 mL tube (Corning, NY, USA) with 9 mL of sterile 1X phosphate saline (PBS) and then vortexed with glass beads (3 mm size) for 3 min. Particles and remains were removed by centrifugation at 700× *g* for 1 min, and the supernatant was collected and centrifuged at 12,000× *g* for 5 min. The pellet was rinsed twice with 1X PBS and kept at −20 °C for DNA extraction.

For DNA extraction, the pellet was mixed with 50 mM EDTA and treated with 10 mg/mL lysozyme (Sigma Aldrich Co., St. Louis, MO, USA) for 45 min at 37 °C. The bacterial genomic DNA was recovered using the Wizard Genomic DNA purification kit (Promega Corp., Madison, WI, USA), following the manufacturer’s instructions.

### 2.10. Cecal Short-Chain Fatty Acids (SCFA) Analysis and Cecal Content pH

As was previously described [28], cecal samples were homogenized in HCl (2 mL, 3%, 1 M), centrifuged and combined with ethyl acetate (100 µL) and acetic acid-d4 (1 µg/mL) before collecting the organic phase to determine short-chain fatty acid (SCFA) composition. Samples were quantified via GC-MS using a TRACE™ 1310 gas chromatograph (Thermo Fisher Scientific, Waltham, MA, USA) and a TraceGOLD™ TG-WaxMS A column (Thermo Fisher Scientific, Waltham, MA, USA). The pH of cecum content was determined using an Oakton^®^ model 700 digital pH meter (Oakton Instruments, Vermon Hills, IL, USA). Before testing, the potentiometer was calibrated with pH buffers at 1.68, 4.01, 7.00, 10.01, and 12.45 according to the manufacturer’s recommendations.

### 2.11. PCR Amplification of Bacterial 16s rDNA

Primers for *Lactobacillus*, *Bifidobacterium*, *E. coli*, and *Clostridium* were designed as previously described [18,22,24,30,47,51]. The universal primers were prepared with the invariant sequence regions in the 16S rRNA of bacteria and used as an internal standard to normalize data. PCR reaction products were isolated by electrophoresis (2% agarose gel), stained with ethidium bromide, and quantified using the Quantity One 1-D analysis software (Bio-Rad, Hercules, CA, USA).

### 2.12. Statistical Analysis

Experimental treatments for the intra-amniotic administration assay were arranged in a completely randomized design and checked for normality of data utilizing the Shapiro–Wilk test before analyzing data further. Once the Gaussian distribution was confirmed, the one-way multiple analysis of variance (ANOVA) was conducted. Differences between treatment groups were compared with a post hoc Duncan test, with results considered statistically different at *p* < 0.05. Statistical analyses were carried out using SPSS version 27.0 software (IBM, Armonk, NY, USA). Results are expressed as mean ± standard error, *n* ≥ 8.

## 3. Results

### 3.1. Fourier Transform Infrared (FTIR) of NRTBCl

**Figure 2:**
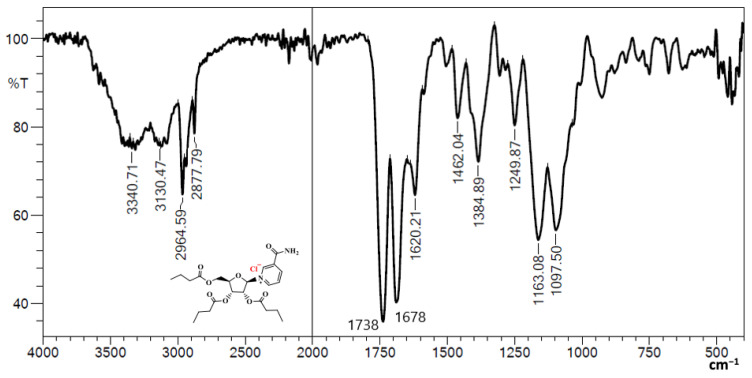
FTIR of NRTBCl.

The FTIR of NRTBCl shows two bands at 3340 and 3130 cm^−1^, which are asymmetric and symmetric stretching bonds of NH_2_ in the amide functional group. The exitance of two bands at 2964 and 2877 cm^−1^ is attributed to asymmetric and symmetric stretching vibrations of aliphatic C-H. A strong band at 1738 cm^−1^ confirms the carbonyl of ester groups in this compound. The carbonyl of the amide functional group appears at 1678 cm^−1^. The band at 1620 cm^−1^ is evidence of the C=C bond in the pyridinium ring. Two bands at 1462 and 1384 cm^−1^ show out-of-plane C-H bending vibrations of the methylene and methyl groups, respectively. The stretching vibrations of the C-O bonds in the ester groups and ribose ring appear at 1163 and 1097 cm^−1^ (Figure 2). The obtained FTIR results confirm the functional groups in the NRTBCl structure.

### 3.2. ^1^H NMR of NRTBCl

**Figure 3:**
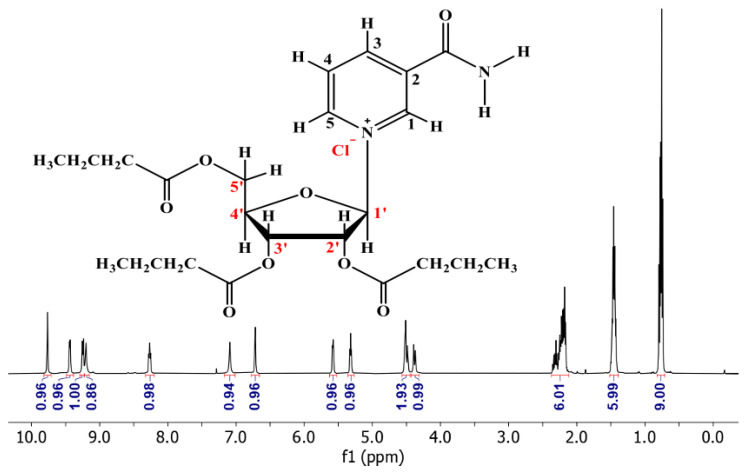
^1^H NMR of NRTBCl in CDCl_3_.

**Figure 4:**
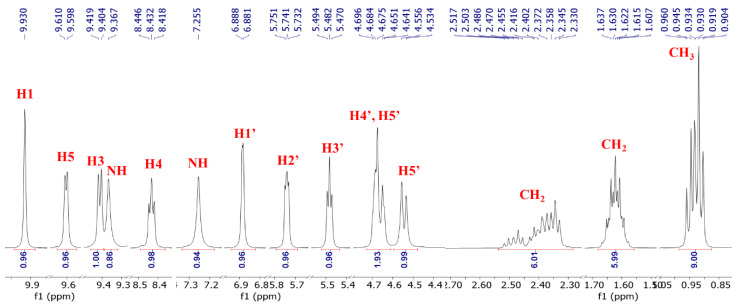
Expanded ^1^H NMR of NRTBCl.

The ^1^H NMR (500 MHz) of NRTBCl was performed in CDCl_3_ at room temperature (Figure 3). The expanded ^1^H NMR of this compound displays that the most deshielded proton (H1) at 9.93 ppm is attributed to the hydrogen located on the pyridinium ring between the positive nitrogen and amide group (Figure 4). A doublet (*J* = 6 Hz) at 9.60 ppm is attributed to H5 located on the pyridinium ring in a position ortho to the positive nitrogen. The chemical shift of H3 in the para position with respect to the positive nitrogen appears at 9.41 ppm as a doublet peak (*J* = 7.5 Hz). Because of the interaction between nitrogen lone pair and carbonyl of the amide group, the chemical shifts of NH_2_ protons are not equivalent in NRTBCl. In this compound, one of the NH_2_ protons appears at 9.37 ppm and another at 7.25 ppm. The final hydrogen on the pyridinium ring is H4 which appears as a triplet peak (*J* = 7 Hz) at 8.43 ppm. In the structure of NRTBCl, there are four hydrogens on the ribose ring. The anomeric hydrogen (H1′) is impacted more by the oxygen atom of the ribose ring and the positive nitrogen of the pyridinium ring so that this hydrogen appears at 6.88 ppm as a doublet peak (*J* = 3.5 Hz). H2′ and H3′ are neighbors and appear as two triplet peaks (*J* = 5 Hz) and (*J* = 6 Hz) with chemical shifts of 5.74 and 5.48 ppm, respectively. Since H2′ is closer to the anomeric center than H3′, its chemical shift is more deshielded than H3′. H4′ in the ribose ring and one of the hydrogens of the methylene group (H5′) bonded to the single oxygen of the ester group overlap and appeared as a multiplet at 4.67 ppm with integral 2. Another hydrogen of this methylene group appears at 4.54 ppm as a doublet (*J* = 11 Hz). In the three chain ester groups of NRTBCl, there are three CH_2_ groups near the ester carbonyl groups, which appear as multiplets between 2.33–2.52 ppm. The multiplet at 1.62 ppm can be attributed to the other three methylene groups near the CH_2_ groups bonded to the carbonyl groups. Finally, a multiplet peak between 0.90–0.96 ppm with an integral of 9 confirms the existence of three methyl groups at the end of the butyrate esters arms. The ^1^H NMR results verified the structure of NRTBCl.

### 3.3. ^13^C NMR of NRTBCl

**Figure 5:**
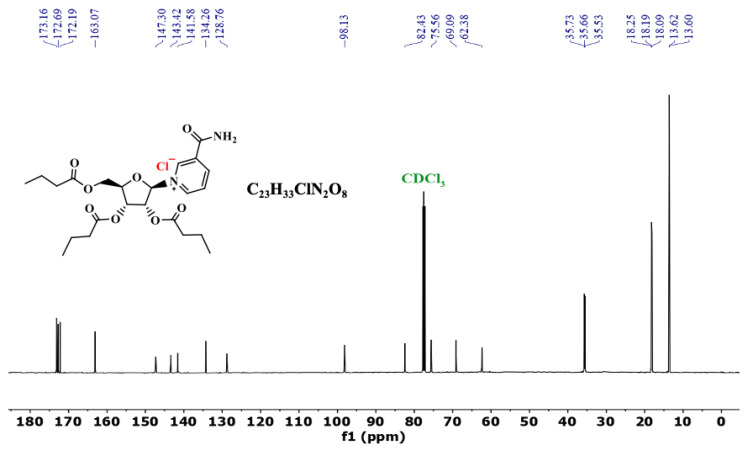
^13^C NMR of NRTBCl in CDCl_3_.

The ^13^C NMR (125 MHz) of NRTBCl in CDCl_3_ was also studied at room temperature (Figure 5). The ^13^C NMR of this compound exhibits three peaks at 173.2, 172.7, and 172.2 ppm, attributed to the three different carbonyl carbons of the ester groups in the structure of NRTBCl. A peak at 163.1 ppm confirms the carbonyl of the amide group in this compound. There are five distinct peaks at 147.3, 143.4, 141.6, 134.3, and 128.8 ppm for the carbons in the pyridinium ring. Four peaks at 98.1, 82.4, 75.6, and 69.1 ppm confirmed the existence of a ribose ring in the structure of NRTBCl, and the chemical shift of the methylene carbon bonded to the single oxygen of the ester group appears at 62.4 ppm. In the three short ester chains of NRTBCl, three distinct peaks at 35.73, 35.66, and 35.5 ppm are attributed to the three CH_2_ groups near the carbonyl carbons of the ester groups (Figure 5). The methylene groups in these chains appear at 18.25, 18.2, and 18.1 ppm. Because the chemical shifts of the methyl groups are very close to each other, one of the methyl groups overlaps with the other one, and these three methyl groups appear as two peaks at 13.62 and 13.60 ppm. The obtained results of ^13^C NMR corroborated well with the ^1^H NMR results to verify the NRTBCl structure.

### 3.4. LC-MS Analysis of NRTBCl

**Figure 6:**
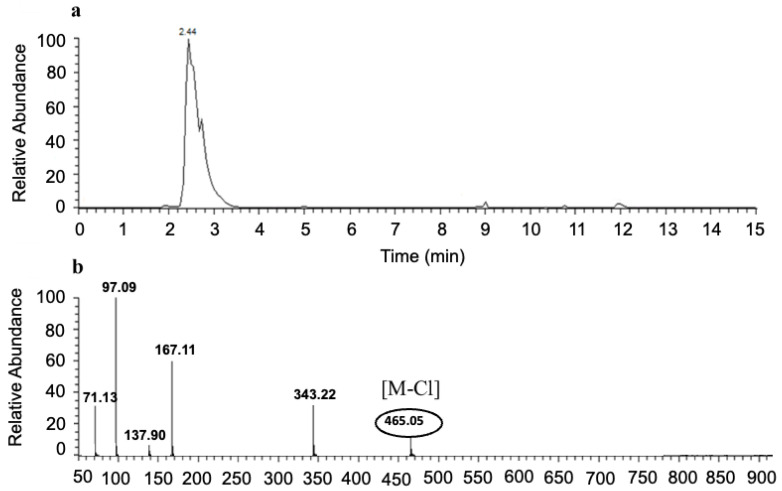
SRM LC-MS of NRTBCl. (**a**) SRM LC of NRTBCl. (**b**) Mass spectrum of NRTBCl.

To confirm the presence of three butyrate groups, LC-MS was performed to find the molecular weight of NRTBCl (Figure 6). The selected reaction monitoring (SRM) results show a single peak with 465.05 m/z (M-Cl) that agrees with the structure of the NRTB cation. Interestingly, a fragment with 343.22 m/z is attributed to the ribose-trioleate molecule formed by eliminating the nicotinamide molecule from NRTBCl.

### 3.5. FTIR of NRTOCl

FTIR (cm^−1^): 3288 (asymmetric stretching vibration of N-H), 3122 (symmetric stretching vibration of N-H), 3005 (stretching vibration of vinyl and aromatic C-H), 2922 (asymmetric stretching vibration of aliphatic C-H), 2852 (symmetric stretching vibration of aliphatic C-H), 1743 (stretching vibration of C=O in ester groups), 1689 (stretching vibration of C=O in the amide group), 1622 (stretching vibration of C=C), 1458 (out-of-plane C-H bending vibrations of CH_2_), 1379 (out-of-plane C-H bending vibrations of CH_3_), 1161 (stretching vibration of C-O), 1116 (stretching vibration of C-O), 914 (out-of-plane C-H bending vibration of aromatic ring), 721 (out-of-plane C-H bending vibration of vinyl groups), 677 and 632 (out-of-plane C-H bending vibration of aromatic ring). (Supporting information, Figure S1 FTIR of NRTOCl). The obtained FTIR results verified the functional groups of NRTOCl.

### 3.6. ^1^H NMR of NRTOCl

^1^H NMR (500 MHz, CDCl_3_) δ (ppm): 10.34 (s, 1 H, pyridinium ring), 9.86 (s, 1 H, NH), 9.44 (d, *J* = 10 Hz, 1 H, pyridinium ring), 9.34 (d, *J* = 10 Hz, 1 H, pyridinium ring), 8.20 (t, *J* = 10 Hz, 1 H, pyridinium ring), 6.75 (d, *J* = 5 Hz, 1 H, ribose ring), 6.28 (s, 1 H, NH), 5.57 (t, *J* = 5 Hz, 1 H, ribose ring), 5.43 (t, *J* = 5 Hz, 1 H, ribose ring), 5.35 (m, 6 H, H-C=C-H groups), 4.70 (m, 2 H, ribose ring and one of the diastereotopic methylene group), 4.50 (dd, *J*_1_ = 14, *J*_2_ = 4 Hz, 1 H, diastereotopic methylene group), 2.37–2.55 (m, 6 H, three CH_2_ groups), 2.02 (m, 12 H, six CH_2_ groups), 1.63 (m, 3 H, three CH_2_ groups), 1.30 (m, 60 H, thirty CH_2_ groups), 0.89 (t, 9 H, three CH_3_ groups) (Supporting information, Figure S2 ^1^H NMR of NRTOCl in CDCl_3_, and Figure S3 Expanded ^1^H NMR of NRTOCl).

### 3.7. ^13^C NMR of NRTOCl

^13^C NMR (125 MHz, CDCl_3_) δ (ppm): 173.1, 172.9 and 172.3 (carbonyl of ester groups), 162.5 (carbonyl of amide group), 146.7, 142.5, 141.8 and 134.6 (pyridinium ring), 130.07, 130.06, 130.04, 129.67 and 129.62 (C=C), 127.9 (pyridinium ring), 98.0, 82.9, 75.8 and 69.1 (ribose ring), 62.2 (diastereotopic methylene), 33.9, 33.8, 33.7, 31.9, 29.8, 29.74, 29.73, 29.72, 29.5, 29.34, 29.32, 29.23, 29.22, 29.16, 29.15, 29.11, 29.10, 29.09, 27.24, 27.18, 24.76, 24.73, 24.6, 22.7 and 14.1 (aliphatic carbons in oleate chains) (Supporting information, Figure S4 ^13^C NMR of Netcool in CDCl_3_, and Figure S5 Expanded ^13^C NMR of NRTOCl). The results of ^1^H NMR and ^13^C NMR confirm the structure of NRTOCl.

### 3.8. LC-MS of NRTOCl

A single peak with 1047.52 m/z (M-Cl) agrees with the structure of the NRTO cation. A fragment with 925.70 m/z is attributed to the ribose-trioleate molecule formed by removing the nicotinamide molecule from NRTOCl (Supporting information, Figure S6 SRM LC-MS of NRTOCl. (a) SRM LC of NRTOCl. (b) Mass spectrum of NRTOCl).

### 3.9. Particle Size and Zeta Potential of NRTOCl

NRTOCl was dispersed in DI water using 1% (*v*/*v*) ethanol as a cosolvent, and the average size and zeta potential of the NRTOCl particles were 192 nm and +65 mV, respectively (Supporting information, Figure S7 Particle size of NRTOCl in DI water containing 1% ethanol).

### 3.10. Gross Physiological Parameters

There were no significant differences in body weight between treatment groups. Compared with the non-injected control, the average cecum weight was significantly increased (*p* < 0.05) with NRCl exposure. When compared with NRCl exposure, NRTBCl exposure resulted in significantly increased (*p* < 0.05) cecum weight. Compared with the non-injected and H_2_O control, no significant differences were found with NRCl and NRTBCl and NRTOCl exposure between cecum: bodyweight ratios (Table 2).

### 3.11. Ceca Bacterial Analysis

**Figure 7:**
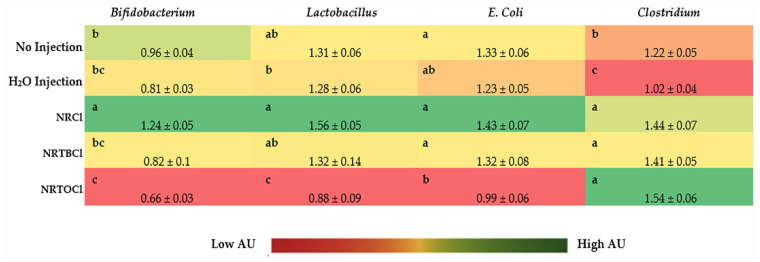
Effects of intra-amniotic administration of experimental solutions on cecal genera and species-level bacterial populations (day of the hatch). Values are means ± SEM, *n* = 10. ^a–c^ Per bacterial category, treatments groups that do not share any letters within the same column are significantly different according to a one-way ANOVA with post hoc Duncan test (*p* < 0.05). AU = arbitrary units.

Cecal genera bacterial populations are shown in Figure 7. NRCl exposure resulted in a significant increase (*p* < 0.05) in the relative abundance of *Bifidobacterium* spp. when compared with all other treatment groups. NRCl derivative exposure did not significantly alter *Bifidobacterium* spp. relative abundance when compared with the controls. *Bifidobacterium* spp. and *Lactobacillus* spp. relative abundance was significantly elevated with NRCl exposure, while NRTOCl exposure decreased relative abundance compared with the H_2_O control (*p* < 0.05). Compared with the non-injected control, NRTOCl treatment resulted in a significant decrease in the relative abundance of *E. coli*. NRCl, NRTBCl, and NTROCl exposure significantly elevated *Clostridium* populations compared with the controls (*p* < 0.05).

### 3.12. Short-Chain Fatty Acids and pH Concentrations in Cecal Contents

**Figure 8:**
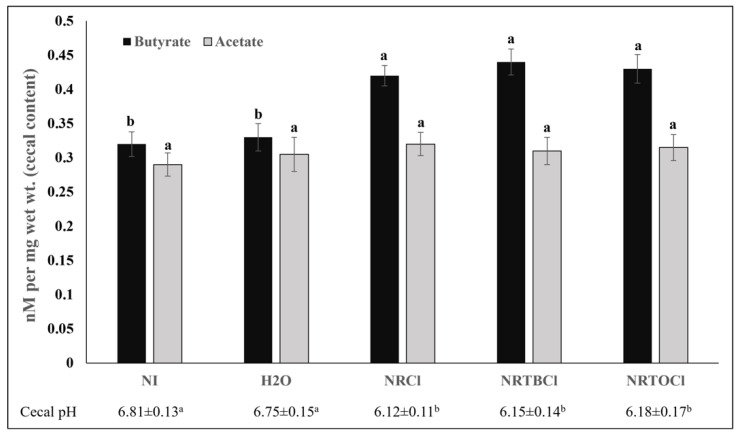
Cecal short-chain fatty acid (SCFA) composition and cecal pH. Values are the means ± SEM, *n* = 5. ^a,b^ Per SCFA (butyrate or acetate) and pH, treatments groups not indicated by the same letter within the same column are significantly different (*p* < 0.05).

Short-chain fatty acid (SCFA) production significantly increased for butyrate, and as a result, cecal chyme pH significantly decreased in NRCl, NRTBCl, and NRTOCl groups compared to the non-injected and water-injected groups (Figure 8).

### 3.13. Duodenal Brush Border Membrane Gene Expression

**Figure 9:**
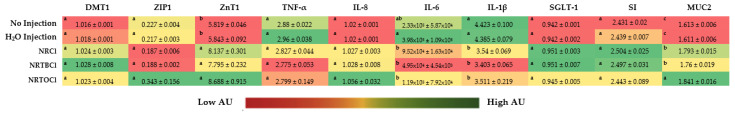
Effects of the intraamniotic administration of experimental solutions on duodenal gene expression. Values are the means ± SEM, *n* = 10. ^a–c^ Per gene, treatments groups not indicated by the same letter within the same column are significantly different (*p* < 0.05). DMT1, Divalent metal transporter 1; ZIP1, Zinc Transport Protein 1; ZnT1, Zinc transporter 1; Sodium/Glucose cotransporter 1; SI, Sucrose isomaltase; MUC2, Mucin 2; TNF-α, Tumor necrosis factor-alpha; IL-8, Interleukin 8; IL-6, Interleukin 6; IL-1β, Interleukin 1 Beta. AU = arbitrary units.

For iron-related protein gene expression, divalent metal transporter 1 (DMT1), there was no significant difference between any treatment groups; however, there was a general trend of increasing expression of the experimental groups (NRCl, NRTBCl, and NTROCl) compared to the controls (non-injected and H_2_O injected groups) (Figure 9). For zinc transporters gene expression, while there were no significant differences in ZIP1 expression, there was a significant increase (*p* < 0.05) in zinc transporter 1 (ZnT1) with NR and NR derivative exposure (NRCl, NRTBCl, and NTROCl) when compared to the non-injected and H_2_O controls.

As for inflammatory gene expression, NR exposure (NRCl, NRTBCl, and NTROCl) did not alter (*p* > 0.05) the expression of TNF-α and IL-8 relative to the non-injected and H_2_O injection groups. However, there was a significant (*p* < 0.05) down-regulation in the expression of IL-6 and IL-1β in the NR experimental groups (NRCl, NRTBCl, and NRTOCl) compared to the H_2_O injected group.

NRCl and NRTBCl exposure increased gene expression of brush border membrane absorptive proteins, sodium-glucose transporter 1 (SGLT-1), and sucrose isomaltase (SI) relative to the non-injected and H_2_O injected groups. Additionally, the digestive viscoelastic gels formed by mucin (MUC2) (protecting the intestinal cells) were altered significantly (*p* < 0.05) in gene expression within NRCl, NRTBCl, and NRTOCl groups compared to the non-injected and water injected groups (Figure 9).

## 4. Discussion

NR was discovered as a form of vitamin B_3_ that can increase NAD(P) levels, as NAD^+^ is utilized as a coenzyme for oxidoreductases and a source of ADP ribosyl group for adding one or more ADP-ribose moieties to a protein [52,53,54]. This study focuses on NR derivatives, NRTBCl and NRTOCl, synthesized using butyric anhydride and oleoyl chloride, respectively. The reactions were executed under mild conditions and resulted in acceptable yields. The final purified products were characterized using FTIR, NMR, and LC-MS to determine their structures.

The effects of synthesized and characterized NRCl, NRTBCl, and NRTOCl as prebiotic supplementation on duodenal brush border molecular and cecal microbial populations were investigated in vivo. These compounds did not show any significant effects on average body weight (Table 2). However, NRCl treatment substantially reduced the average cecum weight compared to the non-injection control. A potential explanation for this observation may be the three butyrate groups that are integral structural parts of NRTBCl, which can be metabolized to butyric acid by the gut microbiota [55,56,57]. Previously, Aghazadeh & TahaYazdi (2012) found that butyric acid dietary supplementation increased the weight of the liver and intestines compared to butyric acid-free diets [56]. Additionally, Panda et al. (2009) demonstrated that 0.4% dietary butyrate supplementation significantly increased (*p* < 0.05) body weight, intestinal tract health, and villi development in vivo (*Gallus gallus*) in comparison to antibiotic-treated and control groups [57].

Further, we studied the effects of the investigated NR compounds on cecum bacterial populations (Figure 7). Significant (*p* < 0.05) increases in *Clostridium* populations were observed with NR exposure compared to the controls, where the *Clostridium* genus houses a well-known butyrate producer, Clostridial cluster XIVa; a microbial cluster that may affect intestinal butyrate levels [58,59,60,61]. As was previously demonstrated, butyrate, a short-chain fatty acid, plays a key role as a significant energy source for gut bacteria and the induction of epithelial cell proliferation, supporting intestinal development and health [28,55,56,57,62,63,64]. In the current study, butyrate cecal contents concentrations were higher in treatment groups relative to controls (*p* < 0.05, Figure 8). However, the observed increased abundance in *Clostridium* class is not a direct indicator of butyrate production. Previously, Lozada-Fernandez et al. (2022) found similar results, demonstrating that NR-treated mice had an increase in fecal propionate, butyrate, valerate and isobutyrate concentrations compared to controls while having an increased population of *Firmicutes* (oxidizing butyrate for growth) [65]. This result was hypothesized to be caused by *Firmicutes* metagenome-assembled genomes (MAGs) utilizing acetyl coenzyme A (acetyl- CoA) butyrate synthesis pathway, thus indicating the NR supplementation enriches butyrate-producing *Firmicutes* (e.g., *Clostridium* sp.) [65]. However, current observations indicate that further and detailed investigation, especially the specific quantification of butyrate-producing bacteria is essential to better understand the microbial basis of an increase in butyrate, as a result of NR consumption. 

The NRCl treatment group demonstrated a significant (*p* < 0.05) increase in *Bifidobacterium*, *Lactobacillus*, and *E. coli* populations compared to the other experimental groups. This may be attributed to the potential and indirect targeting of *Clostridium* due to the immunomodulatory role that nicotinamide holds, as was similarly found under *M. tuberculosis* and HIV infections circumstances [66,67,68,69,70,71]. Interestingly, the impact of NRCl on the increase of the populations of *Bifidobacterium* and *Lactobacillus* was more than that of *E. coli* in comparison to H_2_O, and non-injected controls. Elevated levels of commensal bacterial populations (*Bifidobacterium* and *Lactobacillus*) may be due to NRCl, and its derivatives consumed by beneficial bacteria within the gut. These commensal bacteria are preferred by the host, and this preference decreases the populations of potentially invasive bacteria through the production of antimicrobial defensins and cathelicidins (i.e., muramidase, α-defensins, β-defensins) by the host’s innate immune system in the small intestine [72,73,74,75].

Contrary to NRCl, the NRTBCl treatment did not alter the populations of *Bifidobacterium*, *Lactobacillus*, or *E. coli*. Further, relative to NRCl and NRTBCl, the NRTOCl treatment significantly (*p* < 0.05) reduced the populations of *Bifidobacterium*, *Lactobacillus*, and *E. coli*. Structurally, NRTOCl comprises a quaternary ammonium group with a positive charge and three hydrophobic oleate branches. This structure can act as a cationic surfactant and negatively affect these bacterial populations. Reuerio et al. (2016) demonstrated a significant increase in Bacteroidetes and Firmicutes (i.e., *Lactobacillus*) in subjects with elevated ammonia levels, leading to a decreased population of Actinobacteria (i.e., *Bifidobacterium*), which supports the microbial findings presented here [76]. Therefore, the increase in ammonium associated with supplementation of NRTOCl led to a bacterial profile shift towards Bacteroidetes, possibly lowering the population of *Bifidobacterium*, *Lactobacillus*, and *E. coli*.

Previous studies have demonstrated that the intra-amniotic administration of polyphenols and other dietary substances (as soluble fiber extracts) has affected BBM functionality through alterations in gene expression of key BBM digestive and absorptive proteins [29,45,47,50,77,78]. In the current study, we investigated the effects of NRCl and its derivatives on BBM gene expression (Figure 9), and the results demonstrated that NRCl, NRTBCl, and NRTOCl increased the expression of ZnT1 and MUC2 and decreased the expression of IL-6. In comparison with the other investigated compounds and controls, NRTOCl significantly increased MUC2 gene expression level, which is a valuable factor in maintaining intestinal health, as MUC2 that encodes mucin 2 protein that is secreted onto mucosal surfaces and protects the intestinal epithelium cells where its disruption causes several pathologies [79,80,81]. A consistently high level of MUC2 expression can be associated with an essential protective barrier against external pathogens (as indicated by microbial findings, Figure 7) due to MUC2’s diverse functions in intestinal homeostasis. It was previously demonstrated that there was embryonic development of MUC2 at nine weeks of gestation, making it an important marker for the differentiation of secretory cell lineages [47].

The significant decrease in the gene expression of IL-6, which acts as a pleiotropic pro-inflammatory cytokine, is indirectly activated by the supplementation of NRCl, NRTBCl, and NRTOCl, to protect the host against invasive bacteria, which further explains the reduction in the abundance of specific invasive bacterial populations (*E. coli*). This finding agrees with previous observations by Elhassan et al. (2019), where oral NR supplementation resulted in significantly decreased IL-6 expression, in vivo [82].

Moreover, gene expression of the ZnT1 transporter was significantly increased in NRCl, NRTBCl, and NRTOCl, compared to the control groups; this observation can be linked to the potential antioxidative effect NRCl, which may lead to increased cellular zinc [52,83,84]. Specifically, ZnT1 acts as a rescue agent under excess zinc conditions to export zinc from cellular organelles to the cytosol [85,86]. This finding may indicate increased zinc content within the cellular organelles of the enterocytes due to NR supplementation, resulting in excess zinc being transported via the basolateral membrane [87]. Previously, it was demonstrated that the intra-amniotic administration of zinc-methionine increased ZnT1 expression due to the introduction of additional zinc [87]. Additionally, ZnT1 expression is increased during times of inflammation as a shuttle of zinc content into the plasma for circulation, which coincides with the increase in IL-6 gene expression [88,89,90,91,92].

Therefore, the administration of NRCl and its derivatives triggers altered expression of key BBM genes involved in digestion, and absorption, with additional effects on intestinal microbiota composition and function. Further and as was previously suggested, these functional changes, as demonstrated via gene expression of key BBM proteins (zinc transporter, inflammatory cytokines, absorptive proteins, and mucin), were previously associated with BBM tissue physiological and morphometric alterations, as increased villi size [24,50,93,94,95,96,97]. These alterations may potentially also be associated with increased proliferation of cellular populations that hold essential roles in BBM function, including, enterocytes, and therefore, increased villus surface area (the intestinal digestive and absorptive surface), and goblet cells (produce and secrete mucus), both number and diameter in intestinal villi and crypt [18,22,24,47,50,93,98,99,100,101,102,103,104,105]. In this context, it was previously demonstrated that colonocytes and enterocytes oxidation pathways utilize butyrate as fuel for cell metabolism [106,107,108], via SL16 monocarboxylate transporter 1 (MCT1, SCL16A1) and Sodium-coupled monocarboxylate transporter 1 (SMCT1, SLC5A8) that transport butyrate through epithelial cells in the small intestine [109,110]. This allows butyrate and its metabolites to enter the bloodstream, and to potentially affect anti-inflammatory cytokines [111,112], by mediating the binding of free fatty acid receptors (FFARs) [113,114,115], and by that to indirectly support rapid glycolytic energy extraction for undifferentiated stem cells [116,117,118]. Further, Kien et al. demonstrated in piglets that cecal infu-sion of butyrate significantly (*p* = 0.007) supported small intestinal enterocytes proliferation (ileum and jejunum) [119]. Similarly, Zhang et al. suggested that propionate’s cecal fermentation plays a significant role in jejunum development and gut health [120]. In addition, De Vadder et al. demonstrated that FOS and SCFAs (propionate and butyrate) increased cell proliferation, via FFAR3 receptors in rats [101]. However, it is important to emphasize that further assessments are necessary to confirm the potential effects of NR and its derivatives on intestinal morphology and functionality due to SCFAs production (specifically butyrate) by the cecal and/or small intestinal microbiome.

Overall, current results introduce an innovative approach to evaluating the impact of NR and its derivatives on BBM functional biomarkers, and intestinal microbial populations, in vivo.

## 5. Conclusions

This study is the first to demonstrate the effects of nicotinamide riboside and its derivatives on duodenal BBM gene expression and cecal microbial profiles, in-vivo. We have synthesized and characterized two derivatives of vitamin B_3_–NRTOCl and NRTBCl. Through the in vivo study, we found that NRTOCl has the potential to improve BBM functionality by increasing MUC2, and ZnT1 and reducing the expression of inflammatory cytokine IL-1β. Additionally, we detected significant differences in cecal bacterial populations, which suggests that NR and its derivatives positively modulate the intestinal microbial profile, composition, and function. Further studies are warranted to validate the findings of the current research and establish the safety of the synthesized compounds.

## Figures and Tables

**Table 1:** Real-time polymerase chain reaction (RT-PCR) primer sequences.

Gene	Forward Primer (5′→3′)	Reverse Primer (5′→3′)	Base Pair	GI Identifier
Iron Metabolism				
DMT1	TTGATTCAGAGCCTCCCATTAG	GCGAGGAGTAGGCTTGTATTT	101	206597489
Zinc Metabolism				
ZIP1	TGCCTCAGTTTCCCTCAC	GGCTCTTAAGGGCACTTCT	144	107055139
ZnT1	GGTAACAGAGCTGCCTTAACT	GGTAACAGAGCTGCCTTAACT	105	54109718
Inflammatory Response				
TNF-α	GACAGCCTATGCCAACAAGTA	TTACAGGAAGGGCAACTCATC	109	53854909
IL-8	TCATCCATCCCAAGTTCATTCA	GACACACTTCTCTGCCATCTT	105	395872
IL-6	ACCTCATCCTCCGAGACTTTA	GCACTGAAACTCCTGGTCTT	105	302315692
IL-1β	CTCACAGTCCTTCGACATCTTC	TGTTGAGCCTCACTTTCTGG	119	88702685
BBM functionality				
SGLT-1	GCATCCTTACTCTGTGGTACTG	TATCCGCACATCACACATCC	106	8346783
SI	CCAGCAATGCCAGCATATTG	CGGTTTCTCCTTACCACTTCTT	95	2246388
MUC2	CCTGCTGCAAGGAAGTAGAA	GGAAGATCAGAGTGGTGCATAG	155	423101
18S	GCAAGACGAACTAAAGCGAAAG	TCGGAACTACGACGGTATCT	100	7262899

DMT-1, Divalent metal transporter; ZIP1, Zinc transporter 1; ZnT1, zinc transporter 1; TNF-α, tumor necrosis factor; IL-8, interleukin 8; IL-6, interleukin 6; IL-1β, interleukin 1 beta; SGLT-1, sodium-glucose transporter 1; SI, sucrose isomaltase; Muc2, Mucin 2; 18S rRNA, 18S ribosomal subunit.

**Table 2:** Gross physiological parameters measured on the day of hatch (Day 21) ^1^.

Group Name	Average Body Weight (g)	Average Cecum Weight (g)	CW: BW
NI	42.06 ± 1.35 ^a^	0.45 ± 0.05 ^a^	0.011 ± 0.002 ^a^
H_2_O	42.16 ± 1.28 ^a^	0.29 ± 0.04 ^b^^c^	0.007 ± 0.001 ^a^^b^
NRCl	42.74 ± 1.30 ^a^	0.23 ± 0.05 ^c^	0.005 ± 0.001 ^b^
NRTBCl	43.80 ± 0.74 ^a^	0.38 ± 0.06 ^a^^b^	0.009 ± 0.001 ^a^^b^
NRTOCl	43.17 ± 0.85 ^a^	0.35 ± 0.04 ^a^^b^^c^	0.008 ± 0.001 ^a^^b^

^1^ Values are the means ± SEM, *n* = 10. ^a–c^ Treatment groups not indicated by the same letter within the same column are significantly different (*p* < 0.05). NI = non-injected, CW = cecum weight, BW = body weight.

## Data Availability

The data presented in this study are available on request from the corresponding author.

## Supplementary Materials

The following are available online at https://www.mdpi.com/article/10.3390/nu14153130/s1, Figure S1: FT-IR of NRTOCl, Figure S2: ^1^H NMR of NRTOCl in CDCl_3_, Figure S3: Expanded ^1^H NMR of NRTOCl, Figure S4: ^13^C NMR of NRTOCl in CDCl_3_, Figure S5: Expanded ^13^C NMR of NRTOCl, Figure S6: SRM LC-MS of NRTOCl. (a) SRM LC of NRTOCl. (b) Mass spectrum of NRTOCl, Figure S7: Particle size of NRTOCl in DI water containing 1% ethanol.

## References

[1] Da Silva B.P., Martino H.S.D., Tako E. (2021). Plant Origin Prebiotics Affect Duodenal Brush Border Membrane Functionality and Morphology, in Vivo (Gallus Gallus). Food Funct..

[2] Kataoka K. (2016). The Intestinal Microbiota and Its Role in Human Health and Disease. J. Med. Investig..

[3] Tanes C., Bittinger K., Gao Y., Friedman E.S., Nessel L., Paladhi U.R., Chau L., Panfen E., Fischbach M.A., Braun J. (2021). Role of Dietary Fiber in the Recovery of the Human Gut Microbiome and Its Metabolome. Cell Host Microbe.

[4] Brisbin J.T., Gong J., Sharif S. (2008). Interactions between Commensal Bacteria and the Gut-Associated Immune System of the Chicken. Anim. Health Res. Rev..

[5] Zhang Y.-J., Li S., Gan R.-Y., Zhou T., Xu D.-P., Li H.-B. (2015). Impacts of Gut Bacteria on Human Health and Diseases. Int. J. Mol. Sci..

[6] Reed S., Neuman H., Glahn R.P., Koren O., Tako E. (2017). Characterizing the Gut (Gallus Gallus) Microbiota Following the Consumption of an Iron Biofortified Rwandan Cream Seeded Carioca (Phaseolus Vulgaris L.) Bean-Based Diet. PLoS ONE.

[7] Bouis H.E., Hotz C., McClafferty B., Meenakshi J.V., Pfeiffer W.H. (2011). Biofortification: A New Tool to Reduce Micronutrient Malnutrition. Food Nutr. Bull..

[8] Welch R.M. (2005). Biotechnology, Biofortification, and Global Health. Food Nutr. Bull..

[9] Mayer J.E., Pfeiffer W.H., Beyer P. (2008). Biofortified Crops to Alleviate Micronutrient Malnutrition. Curr. Opin. Plant Biol..

[10] Juste Contin Gomes M., Stampini Duarte Martino H., Tako E. (2021). Effects of Iron and Zinc Biofortified Foods on Gut Microbiota in Vivo (*Gallus gallus*): A Systematic Review. Nutrients.

[11] Tian L., Tan Y., Chen G., Wang G., Sun J., Ou S., Chen W., Bai W. (2019). Metabolism of Anthocyanins and Consequent Effects on the Gut Microbiota. Crit. Rev. Food Sci. Nutr..

[12] Gibson G.R., Scott K.P., Rastall R.A., Tuohy K.M., Hotchkiss A., Dubert-Ferrandon A., Gareau M., Murphy E.F., Saulnier D., Loh G. (2010). Dietary Prebiotics: Current Status and New Definition. Food Sci. Technol. Bull. Funct. Foods.

[13] van der Beek C.M., Canfora E.E., Kip A.M., Gorissen S.H., Damink S.W.O., van Eijk H.M., Holst J.J., Blaak E.E., Dejong C.H., Lenaerts K. (2018). The Prebiotic Inulin Improves Substrate Metabolism and Promotes Short-Chain Fatty Acid Production in Overweight to Obese Men. Metabolism.

[14] McLoughlin R.F., Berthon B.S., Jensen M.E., Baines K.J., Wood L.G. (2017). Short-Chain Fatty Acids, Prebiotics, Synbiotics, and Systemic Inflammation: A Systematic Review and Meta-Analysis. Am. J. Clin. Nutr..

[15] Preidis G.A., Versalovic J. (2009). Targeting the Human Microbiome with Antibiotics, Probiotics, and Prebiotics: Gastroenterology Enters the Metagenomics Era. Gastroenterology.

[16] Verediano T.A., Stampini Duarte Martino H., Dias Paes M.C., Tako E. (2021). Effects of Anthocyanin on Intestinal Health: A Systematic Review. Nutrients.

[17] Hou T., Tako E. (2018). The in Ovo Feeding Administration (Gallus Gallus)—An Emerging in Vivo Approach to Assess Bioactive Compounds with Potential Nutritional Benefits. Nutrients.

[18] Wiesinger J.A., Glahn R.P., Cichy K.A., Kolba N., Hart J.J., Tako E. (2019). An in Vivo (*Gallus gallus*) Feeding Trial Demonstrating the Enhanced Iron Bioavailability Properties of the Fast Cooking Manteca Yellow Bean (*Phaseolus vulgaris* L.). Nutrients.

[19] Knez M., Tako E., Glahn R.P., Kolba N., de Courcy-Ireland E., Stangoulis J.C. (2018). Linoleic Acid: Dihomo-γ-Linolenic Acid Ratio Predicts the Efficacy of Zn-Biofortified Wheat in Chicken (*Gallus gallus*). J. Agric. Food Chem..

[20] Dias D.M., Kolba N., Binyamin D., Ziv O., Regini Nutti M., Martino H.S.D., Glahn R.P., Koren O., Tako E. (2018). Iron Biofortified Carioca Bean (*Phaseolus vulgaris* L.)—Based Brazilian Diet Delivers More Absorbable Iron and Affects the Gut Microbiota in Vivo (*Gallus gallus*). Nutrients.

[21] Tako E., Bar H., Glahn R.P. (2016). The Combined Application of the Caco-2 Cell Bioassay Coupled with in Vivo (Gallus Gallus) Feeding Trial Represents an Effective Approach to Predicting Fe Bioavailability in Humans. Nutrients.

[22] Agrizzi Verediano T., Stampini Duarte Martino H., Kolba N., Fu Y., Cristina Dias Paes M., Tako E. (2022). Black Corn (*Zea mays* L.) Soluble Extract Showed Anti-Inflammatory Effects and Improved the Intestinal Barrier Integrity in Vivo (*Gallus gallus*). Food Res. Int..

[23] Agrizzi Verediano T., Agarwal N., Gomes M.J.C., Duarte Martino H.S., Tako E. (2022). Effects of Dietary Fiber on Intestinal Iron Absorption, and Physiological Status: A Systematic Review of in Vivo and Clinical Studies. Crit. Rev. Food Sci. Nutr..

[24] Gomes M.J.C., Martino H.S.D., Kolba N., Cheng J., Agarwal N., De Moura Rocha M., Tako E. (2022). Zinc Biofortified Cowpea (*Vigna unguiculata* L. Walp.) Soluble Extracts Modulate Assessed Cecal Bacterial Populations and Gut Morphology in Vivo (*Gallus gallus*). Front. BioScience Landmark.

[25] Uni Z., Ferket P.R., Tako E., Kedar O. (2005). In Ovo Feeding Improves Energy Status of Late-Term Chicken Embryos. Poult. Sci..

[26] Liu H.H., Wang J.W., Chen X., Zhang R.P., Yu H.Y., Jin H.B., Li L., Han C.C. (2011). In Ovo Administration of RhIGF-1 to Duck Eggs Affects the Expression of Myogenic Transcription Factors and Muscle Mass during Late Embryo Development. J. Appl. Physiol..

[27] Selim S.A., Gaafar K.M., El-ballal S.S. (2012). Influence of In-Ovo Administration with Vitamin E and Ascorbic Acid on Theperformance of Muscovy Ducks. Emir. J. Food Agric..

[28] Beasley J.T., Johnson A.A., Kolba N., Bonneau J.P., Glahn R.P., Ozeri L., Koren O., Tako E. (2020). Nicotianamine-Chelated Iron Positively Affects Iron Status, Intestinal Morphology and Microbial Populations in Vivo (*Gallus gallus*). Sci. Rep..

[29] Carboni J., Reed S., Kolba N., Eshel A., Koren O., Tako E. (2020). Alterations in the Intestinal Morphology, Gut Microbiota, and Trace Mineral Status Following Intra-Amniotic Administration (*Gallus gallus*) of Teff (*Eragrostis tef*) Seed Extracts. Nutrients.

[30] Pereira da Silva B., Kolba N., Duarte Martino H.S., Hart J.J., Tako E. (2019). Soluble Extracts from Chia Seed (*Salvia hispanica* L.) Affect Brush Border Membrane Functionality, Morphology and Intestinal Bacterial Populations In Vivo (*Gallus gallus*). Nutrients.

[31] Martens C.R., Denman B.A., Mazzo M.R., Armstrong M.L., Reisdorph N., McQueen M.B., Chonchol M., Seals D.R. (2018). Chronic Nicotinamide Riboside Supplementation Is Well-Tolerated and Elevates NAD+ in Healthy Middle-Aged and Older Adults. Nat. Commun..

[32] Yang T., Chan N.Y.-K., Sauve A.A. (2007). Syntheses of Nicotinamide Riboside and Derivatives: Effective Agents for Increasing Nicotinamide Adenine Dinucleotide Concentrations in Mammalian Cells. J. Med. Chem..

[33] Braidy N., Berg J., Clement J., Khorshidi F., Poljak A., Jayasena T., Grant R., Sachdev P. (2019). Role of Nicotinamide Adenine Dinucleotide and Related Precursors as Therapeutic Targets for Age-Related Degenerative Diseases: Rationale, Biochemistry, Pharmacokinetics, and Outcomes. Antioxid. Redox Signal..

[34] Gonzalez J.M., Jackson A.R. (2020). In Ovo Feeding of Nicotinamide Riboside Affects Broiler Pectoralis Major Muscle Development. Transl. Anim. Sci..

[35] Zhang H., Ryu D., Wu Y., Gariani K., Wang X., Luan P., D’Amico D., Ropelle E.R., Lutolf M.P., Aebersold R. (2016). NAD+ Repletion Improves Mitochondrial and Stem Cell Function and Enhances Life Span in Mice. Science.

[36] Cerutti R., Pirinen E., Lamperti C., Marchet S., Sauve A.A., Li W., Leoni V., Schon E.A., Dantzer F., Auwerx J. (2014). NAD+-Dependent Activation of Sirt1 Corrects the Phenotype in a Mouse Model of Mitochondrial Disease. Cell Metab..

[37] Gong B., Pan Y., Vempati P., Zhao W., Knable L., Ho L., Wang J., Sastre M., Ono K., Sauve A.A. (2013). Nicotinamide Riboside Restores Cognition through an Upregulation of Proliferator-Activated Receptor-γ Coactivator 1α Regulated β-Secretase 1 Degradation and Mitochondrial Gene Expression in Alzheimer’s Mouse Models. Neurobiol. Aging.

[38] Cantó C., Houtkooper R.H., Pirinen E., Youn D.Y., Oosterveer M.H., Cen Y., Fernandez-Marcos P.J., Yamamoto H., Andreux P.A., Cettour-Rose P. (2012). The NAD+ Precursor Nicotinamide Riboside Enhances Oxidative Metabolism and Protects against High-Fat Diet-Induced Obesity. Cell Metab..

[39] Dollerup O.L., Christensen B., Svart M., Schmidt M.S., Sulek K., Ringgaard S., Stødkilde-Jørgensen H., Møller N., Brenner C., Treebak J.T. (2018). A Randomized Placebo-Controlled Clinical Trial of Nicotinamide Riboside in Obese Men: Safety, Insulin-Sensitivity, and Lipid-Mobilizing Effects. Am. J. Clin. Nutr..

[40] Lee H.J., Hong Y.-S., Jun W., Yang S.J. (2015). Nicotinamide Riboside Ameliorates Hepatic Metaflammation by Modulating NLRP3 Inflammasome in a Rodent Model of Type 2 Diabetes. J. Med. Food.

[41] Mehmel M., Jovanović N., Spitz U. (2020). Nicotinamide Riboside—the Current State of Research and Therapeutic Uses. Nutrients.

[42] Zhou Y., Fu B., Zheng X., Wang D., Zhao C., Qi Y., Sun R., Tian Z., Xu X., Wei H. (2020). Pathogenic T-Cells and Inflammatory Monocytes Incite Inflammatory Storms in Severe COVID-19 Patients. Natl. Sci. Rev..

[43] Campbell M.T., Jones D.S., Andrews G.P., Li S. (2019). Understanding the Physicochemical Properties and Degradation Kinetics of Nicotinamide Riboside, a Promising Vitamin B3nutritional Supplement. Food Nutr. Res..

[44] Xu X., Jackson A.R., Gonzalez J.M. (2021). The Effects of in Ovo Nicotinamide Riboside Dose on Broiler Myogenesis. Poult. Sci..

[45] Pacifici S., Song J., Zhang C., Wang Q., Glahn R., Kolba N., Tako E. (2017). Intra Amniotic Administration of Raffinose and Stachyose Affects the Intestinal Brush Border Functionality and Alters Gut Microflora Populations. Nutrients.

[46] Tako E. (2020). Dietary Plant-Origin Bio-Active Compounds, Intestinal Functionality, and Microbiome. Nutrients.

[47] Martino H.S.D., Kolba N., Tako E. (2020). Yacon (Smallanthus Sonchifolius) Flour Soluble Extract Improve Intestinal Bacterial Populations, Brush Border Membrane Functionality and Morphology in Vivo (*Gallus gallus*). Food Res. Int..

[48] Tako E., Ferket P.R., Uni Z. (2004). Effects of in Ovo Feeding of Carbohydrates and Beta-Hydroxy-Beta-Methylbutyrate on the Development of Chicken Intestine. Poult. Sci..

[49] Tako E., Glahn R.P., Knez M., Stangoulis J.C. (2014). The Effect of Wheat Prebiotics on the Gut Bacterial Population and Iron Status of Iron Deficient Broiler Chickens. Nutr. J..

[50] Gomes M.J.C., Kolba N., Agarwal N., Kim D., Eshel A., Koren O., Tako E. (2021). Modifications in the Intestinal Functionality, Morphology and Microbiome Following Intra-Amniotic Administration (*Gallus gallus*) of Grape (*Vitis vinifera*) Stilbenes (Resveratrol and Pterostilbene). Nutrients.

[51] Zhu X.Y., Zhong T., Pandya Y., Joerger R.D. (2002). 16S RRNA-Based Analysis of Microbiota from the Cecum of Broiler Chickens. Appl. Environ. Microbiol..

[52] Kourtzidis I.A., Dolopikou C.F., Tsiftsis A.N., Margaritelis N.V., Theodorou A.A., Zervos I.A., Tsantarliotou M.P., Veskoukis A.S., Vrabas I.S., Paschalis V. (2018). Nicotinamide Riboside Supplementation Dysregulates Redox and Energy Metabolism in Rats: Implications for Exercise Performance. Exp. Physiol..

[53] Bieganowski P., Brenner C. (2004). Discoveries of Nicotinamide Riboside as a Nutrient and Conserved NRK Genes Establish a Preiss-Handler Independent Route to NAD+ in Fungi and Humans. Cell.

[54] Bürkle A. (2001). Physiology and Pathophysiology of Poly(ADP-Ribosyl)Ation *: Review Articles. Bioessays.

[55] Chen R., Xu Y., Wu P., Zhou H., Lasanajak Y., Fang Y., Tang L., Ye L., Li X., Cai Z. (2019). Transplantation of Fecal Microbiota Rich in Short Chain Fatty Acids and Butyric Acid Treat Cerebral Ischemic Stroke by Regulating Gut Microbiota. Pharmacol. Res..

[56] Aghazadeh A., TahaYazdi M. (2012). Effect of Butyric Acid Supplementation and Whole Wheat Inclusion on the Performance and Carcass Traits of Broilers. SA J. An. Sci..

[57] Panda A.K., Rao S.V.R., Raju M.V.L.N., Sunder G.S. (2009). Effect of Butyric Acid on Performance, Gastrointestinal Tract Health and Carcass Characteristics in Broiler Chickens. Asian Australas. J. Anim. Sci.

[58] Guo P., Zhang K., Ma X., He P. (2020). Clostridium Species as Probiotics: Potentials and Challenges. J. Anim. Sci. Biotechnol..

[59] Van den Abbeele P., Belzer C., Goossens M., Kleerebezem M., De Vos W.M., Thas O., De Weirdt R., Kerckhof F.-M., Van de Wiele T. (2013). Butyrate-Producing Clostridium Cluster XIVa Species Specifically Colonize Mucins in an in Vitro Gut Model. ISME J.

[60] Lopetuso L.R., Scaldaferri F., Petito V., Gasbarrini A. (2013). Commensal Clostridia: Leading Players in the Maintenance of Gut Homeostasis. Gut Pathog..

[61] Wu C.-S., Muthyala S.D.V., Klemashevich C., Ufondu A.U., Menon R., Chen Z., Devaraj S., Jayaraman A., Sun Y. (2021). Age-Dependent Remodeling of Gut Microbiome and Host Serum Metabolome in Mice. Aging.

[62] Kaczmarek S.A., Barri A., Hejdysz M., Rutkowski A. (2016). Effect of Different Doses of Coated Butyric Acid on Growth Performance and Energy Utilization in Broilers. Poult. Sci..

[63] Zou X., Ji J., Qu H., Wang J., Shu D.M., Wang Y., Liu T.F., Li Y., Luo C.L. (2019). Effects of Sodium Butyrate on Intestinal Health and Gut Microbiota Composition during Intestinal Inflammation Progression in Broilers. Poult. Sci..

[64] Aalamifar H., Soltanian S., Vazirzadeh A., Akhlaghi M., Morshedi V., Gholamhosseini A., Torfi Mozanzadeh M. (2020). Dietary Butyric Acid Improved Growth, Digestive Enzyme Activities and Humoral Immune Parameters in Barramundi (*Lates Calcarifer*). Aquacult. Nutr..

[65] Lozada-Fernández V.V., de Leon O., Kellogg S.L., Saravia F.L., Hadiono M.A., Atkinson S.N., Grobe J.L., Kirby J.R. (2022). Nicotinamide Riboside-Conditioned Microbiota Deflects High-Fat Diet-Induced Weight Gain in Mice. mSystems.

[66] Schein P.S., Loftus S. (1968). Streptozotocin: Depression of Mouse Liver Pyridine Nucleotides. Cancer Res..

[67] Nagai A., Matsumiya H., Hayashi M., Yasui S., Okamoto H., Konno K. (1994). Effects of Nicotinamide and Niacin on Bleomycin-Induced Acute Injury and Subsequent Fibrosis in Hamster Lungs. Exp. Lung Res..

[68] LeClaire R.D., Kell W., Bavari S., Smith T.J., Hunt R.E. (1996). Protective Effects of Niacinamide in Staphylococcal Enterotoxin-B-Induced Toxicity. Toxicology.

[69] Hiromatsu Y., Sato M., Yamada K., Nonaka K. (1992). Inhibitory Effects of Nicotinamide on Recombinant Human Interferon-Gamma-Induced Intercellular Adhesion Molecule-1 (ICAM-1) and HLA-DR Antigen Expression on Cultured Human Endothelial Cells. Immunol. Lett..

[70] Otsuka A., Hanafusa T., Miyagawa J.-I., Kono N., Tarui S. (1991). Nicotinamide and 3-Aminobenzamide Reduce Interferon-γ -Induced Class II MHC (HLA-DR and -DP) Molecule Expression on Cultured Human Endothelial Cells and Fibroblasts. Immunopharmacol. Immunotoxicol..

[71] Murray M.F. (2003). Nicotinamide: An Oral Antimicrobial Agent with Activity against Both Mycobacterium Tuberculosis and Human Immunodeficiency Virus. Clin. Infect. Dis..

[72] Yang D., Chertov O., Oppenheim J.J. (2001). Participation of Mammalian Defensins and Cathelicidins in Anti-Microbial Immunity: Receptors and Activities of Humandefensins and Cathelicidin (LL-37). J. Leukoc. Biol..

[73] Baquero F., Nombela C. (2012). The Microbiome as a Human Organ. Clin. Microbiol. Infect..

[74] Biesalski H.K. (2016). Nutrition Meets the Microbiome: Micronutrients and the Microbiota: Nutrition Meets the Microbiome. Ann. N. Y. Acad. Sci..

[75] Takakuwa A., Nakamura K., Kikuchi M., Sugimoto R., Ohira S., Yokoi Y., Ayabe T. (2019). Butyric Acid and Leucine Induce α-Defensin Secretion from Small Intestinal Paneth Cells. Nutrients.

[76] Regueiro L., Carballa M., Lema J.M. (2016). Microbiome Response to Controlled Shifts in Ammonium and LCFA Levels in Co-Digestion Systems. J. Biotechnol..

[77] Limage R., Tako E., Kolba N., Guo Z., García-Rodríguez A., Marques C.N.H., Mahler G.J. (2020). TiO_2_ Nanoparticles and Commensal Bacteria Alter Mucus Layer Thickness and Composition in a Gastrointestinal Tract Model. Small.

[78] Kolba N., Guo Z., Olivas F.M., Mahler G.J., Tako E. (2019). Intra-Amniotic Administration (*Gallus gallus*) of TiO_2_, SiO_2_, and ZnO Nanoparticles Affect Brush Border Membrane Functionality and Alters Gut Microflora Populations. Food Chem. Toxicol..

[79] Allen A., Hutton D.A., Pearson J.P. (1998). The MUC2 Gene Product: A Human Intestinal Mucin. Int. J. Biochem. Cell Biol..

[80] Bergstrom K.S.B., Kissoon-Singh V., Gibson D.L., Ma C., Montero M., Sham H.P., Ryz N., Huang T., Velcich A., Finlay B.B. (2010). Muc2 Protects against Lethal Infectious Colitis by Disassociating Pathogenic and Commensal Bacteria from the Colonic Mucosa. PLoS Pathog.

[81] Zarepour M., Bhullar K., Montero M., Ma C., Huang T., Velcich A., Xia L., Vallance B.A. (2013). The Mucin Muc2 Limits Pathogen Burdens and Epithelial Barrier Dysfunction during Salmonella Enterica Serovar Typhimurium Colitis. Infect. Immun..

[82] Elhassan Y.S., Kluckova K., Fletcher R.S., Schmidt M.S., Garten A., Doig C.L., Cartwright D.M., Oakey L., Burley C.V., Jenkinson N. (2019). Nicotinamide Riboside Augments the Aged Human Skeletal Muscle NAD+ Metabolome and Induces Transcriptomic and Anti-Inflammatory Signatures. Cell Rep..

[83] Dolopikou C.F., Kourtzidis I.A., Margaritelis N.V., Vrabas I.S., Koidou I., Kyparos A., Theodorou A.A., Paschalis V., Nikolaidis M.G. (2020). Acute Nicotinamide Riboside Supplementation Improves Redox Homeostasis and Exercise Performance in Old Individuals: A Double-Blind Cross-over Study. Eur. J. Nutr..

[84] Carrera-Juliá S., Moreno M.L., Barrios C., de la Rubia Ortí J.E., Drehmer E. (2020). Antioxidant Alternatives in the Treatment of Amyotrophic Lateral Sclerosis: A Comprehensive Review. Front. Physiol..

[85] Andrews G.K., Wang H., Dey S.K., Palmiter R.D. (2004). Mouse Zinc Transporter 1 Gene Provides an Essential Function during Early Embryonic Development. genesis.

[86] Segal D., Ohana E., Besser L., Hershfinkel M., Moran A., Sekler I. (2004). A Role for ZnT-1 in Regulating Cellular Cation Influx. Biochem. Biophys. Res. Commun..

[87] Tako E., Ferket P., Uni Z. (2005). Changes in Chicken Intestinal Zinc Exporter MRNA Expression and Small Intestinal Functionality Following Intra-Amniotic Zinc-Methionine Administration. J. Nutr. Biochem..

[88] Ghashut R.A., McMillan D.C., Kinsella J., Vasilaki A.T., Talwar D., Duncan A. (2016). The Effect of the Systemic Inflammatory Response on Plasma Zinc and Selenium Adjusted for Albumin. Clin. Nutr..

[89] Vasto S., Mocchegiani E., Candore G., Listì F., Colonna-Romano G., Lio D., Malavolta M., Giacconi R., Cipriano C., Caruso C. (2006). Inflammation, Genes and Zinc in Ageing and Age-Related Diseases. Biogerontology.

[90] Vasto S., Mocchegiani E., Malavolta M., Cuppari I., Listi F., Nuzzo D., Ditta V., Candore G., Caruso C. (2007). Zinc and Inflammatory/Immune Response in Aging. Ann. N. Y. Acad. Sci..

[91] Mburu A.S.W., Thurnham D.I., Mwaniki D.L., Muniu E.M., Alumasa F.M. (2010). The Influence of Inflammation on Plasma Zinc Concentration in Apparently Healthy, HIV+ Kenyan Adults and Zinc Responses after a Multi-Micronutrient Supplement. Eur. J. Clin. Nutr..

[92] McDonald C.M., Suchdev P.S., Krebs N.F., Hess S.Y., Wessells K.R., Ismaily S., Rahman S., Wieringa F.T., Williams A.M., Brown K.H. (2020). Adjusting Plasma or Serum Zinc Concentrations for Inflammation: Biomarkers Reflecting Inflammation and Nutritional Determinants of Anemia (BRINDA) Project. Am. J. Clin. Nutr..

[93] Agarwal N., Kolba N., Jung Y., Cheng J., Tako E. (2022). Saffron (Crocus Sativus L.) Flower Water Extract Disrupts the Cecal Microbiome, Brush Border Membrane Functionality, and Morphology In Vivo (Gallus Gallus). Nutrients.

[94] Agarwal N., Kolba N., Khen N., Even C., Turjeman S., Koren O., Tako E. (2022). Quinoa Soluble Fiber and Quercetin Alter the Composition of the Gut Microbiome and Improve Brush Border Membrane Morphology In Vivo (Gallus Gallus). Nutrients.

[95] Salam S., Iqbal Z., Khan A.A., Mahmood R. (2021). Oral Administration of Thiram Inhibits Brush Border Membrane Enzymes, Oxidizes Proteins and Thiols, Impairs Redox System and Causes Histological Changes in Rat Intestine: A Dose Dependent Study. Pestic. Biochem. Physiol..

[96] Lucea S., Guillén N., Sosa C., Sorribas V. (2022). Inhibition of Phosphate Transport by NAD+/NADH in Brush Border Membrane Vesicles. Am. J. Physiol.-Cell Physiol..

[97] Shahid F., Farooqui Z., Khan A.A., Khan F. (2018). Oral Nigella Sativa Oil and Thymoquinone Administration Ameliorates the Effect of Long-Term Cisplatin Treatment on the Enzymes of Carbohydrate Metabolism, Brush Border Membrane, and Antioxidant Defense in Rat Intestine. Naunyn-Schmiedeberg’s Arch Pharm..

[98] Li H., Cheng J., Yuan Y., Luo R., Zhu Z. (2020). Age-related Intestinal Monosaccharides Transporters Expression and Villus Surface Area Increase in Broiler and Layer Chickens. J. Anim. Physiol. Anim. Nutr..

[99] Coméra C., Cartier C., Gaultier E., Catrice O., Panouille Q., El Hamdi S., Tirez K., Nelissen I., Théodorou V., Houdeau E. (2020). Jejunal Villus Absorption and Paracellular Tight Junction Permeability Are Major Routes for Early Intestinal Uptake of Food-Grade TiO2 Particles: An in Vivo and Ex Vivo Study in Mice. Part Fibre Toxicol..

[100] Tysoe O. (2021). Dietary Fructose Acts on Gut to Increase Nutrient Uptake. Nat. Rev. Endocrinol..

[101] De Vadder F., Kovatcheva-Datchary P., Goncalves D., Vinera J., Zitoun C., Duchampt A., Bäckhed F., Mithieux G. (2014). Microbiota-Generated Metabolites Promote Metabolic Benefits via Gut-Brain Neural Circuits. Cell.

[102] Kelly D. (2008). Regulation of Gut Function and Immunity. Formula for the Future: Nutrition or Pathology? Evaluating Performance and Health in Pigs and Poultry.

[103] Snel J., Harmsen H., van der Wielen P., Williams B. (2002). Dietary Strategies to Influence the Gastrointestinal Microflora of Young Animals, and Its Potential to Improve Intestinal Health. Nutrition and Health in the Gastrointestinal Tract.

[104] Lewis A.J., Southern L.L. (2000). Intestinal Bacteria and Their Influence on Swine Growth. Swine Nutrition.

[105] Apajalahti J., Kettunen A., Graham H. (2004). Characteristics of the Gastrointestinal Microbial Communities, with Special Reference to the Chicken. World’s Poult. Sci. J..

[106] He W., Wu G. (2022). Oxidation of Amino Acids, Glucose, and Fatty Acids as Metabolic Fuels in Enterocytes of Developing Pigs. Amino Acids.

[107] Litvak Y., Byndloss M.X., Bäumler A.J. (2018). Colonocyte Metabolism Shapes the Gut Microbiota. Science.

[108] Salvi P.S., Cowles R.A. (2021). Butyrate and the Intestinal Epithelium: Modulation of Proliferation and Inflammation in Homeostasis and Disease. Cells.

[109] Borthakur A., Saksena S., Gill R.K., Alrefai W.A., Ramaswamy K., Dudeja P.K. (2008). Regulation of Monocarboxylate Transporter 1 (MCT1) Promoter by Butyrate in Human Intestinal Epithelial Cells: Involvement of NF-ΚB Pathway. J. Cell. Biochem..

[110] Gonçalves P., Araújo J.R., Martel F. (2011). Characterization of Butyrate Uptake by Nontransformed Intestinal Epithelial Cell Lines. J Membr. Biol.

[111] Kim M.H., Kang S.G., Park J.H., Yanagisawa M., Kim C.H. (2013). Short-Chain Fatty Acids Activate GPR41 and GPR43 on Intestinal Epithelial Cells to Promote Inflammatory Responses in Mice. Gastroenterology.

[112] Tazoe H., Otomo Y., Kaji I., Tanaka R., Karaki S.-I., Kuwahara A. (2008). Roles of Short-Chain Fatty Acids Receptors, GPR41 and GPR43 on Colonic Functions. J. Physiol. Pharm..

[113] Zhao Y., Chen F., Wu W., Sun M., Bilotta A.J., Yao S., Xiao Y., Huang X., Eaves-Pyles T.D., Golovko G. (2018). GPR43 Mediates Microbiota Metabolite SCFA Regulation of Antimicrobial Peptide Expression in Intestinal Epithelial Cells via Activation of MTOR and STAT3. Mucosal. Immunol..

[114] Fujiwara H., Docampo M.D., Riwes M., Peltier D., Toubai T., Henig I., Wu S.J., Kim S., Taylor A., Brabbs S. (2018). Microbial Metabolite Sensor GPR43 Controls Severity of Experimental GVHD. Nat. Commun..

[115] Brown A.J., Goldsworthy S.M., Barnes A.A., Eilert M.M., Tcheang L., Daniels D., Muir A.I., Wigglesworth M.J., Kinghorn I., Fraser N.J. (2003). The Orphan G Protein-Coupled Receptors GPR41 and GPR43 Are Activated by Propionate and Other Short Chain Carboxylic Acids. J. Biol. Chem..

[116] Barker N., van Es J.H., Kuipers J., Kujala P., van den Born M., Cozijnsen M., Haegebarth A., Korving J., Begthel H., Peters P.J. (2007). Identification of Stem Cells in Small Intestine and Colon by Marker Gene Lgr5. Nature.

[117] Kaiko G.E., Ryu S.H., Koues O.I., Collins P.L., Solnica-Krezel L., Pearce E.J., Pearce E.L., Oltz E.M., Stappenbeck T.S. (2016). The Colonic Crypt Protects Stem Cells from Microbiota-Derived Metabolites. Cell.

[118] Neelis E., Koning B., Rings E., Wijnen R., Nichols B., Hulst J., Gerasimidis K. (2019). The Gut Microbiome in Patients with Intestinal Failure: Current Evidence and Implications for Clinical Practice. J. Parenter. Enter. Nutr..

[119] Kien C.L., Blauwiekel R., Bunn J.Y., Jetton T.L., Frankel W.L., Holst J.J. (2007). Cecal Infusion of Butyrate Increases Intestinal Cell Proliferation in Piglets. J. Nutr..

[120] Zhang Y., Chen H., Zhu W., Yu K. (2019). Cecal Infusion of Sodium Propionate Promotes Intestinal Development and Jejunal Barrier Function in Growing Pigs. Animals.

