# Supplementary material for: Alterations in Intestinal Brush Border Membrane Functionality and Bacterial Populations Following Intra-Amniotic Administration (Gallus gallus) of Nicotinamide Riboside and Its Derivatives
Source: Nutrients. 2022 Jul 29;14(15):3130. doi: 10.3390/nu14153130 (PMC9370700; doi:10.3390/nu14153130)
Supplement: Supplementary file 1 [file nutrients-14-03130-s001.zip › nutrients-1722599-supplementary.pdf]

Supporting Information

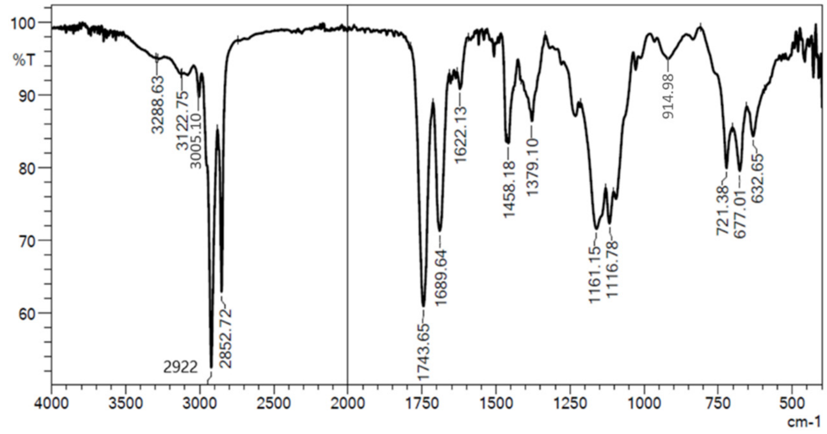

Figure S1. FTIR of NRTOCl.

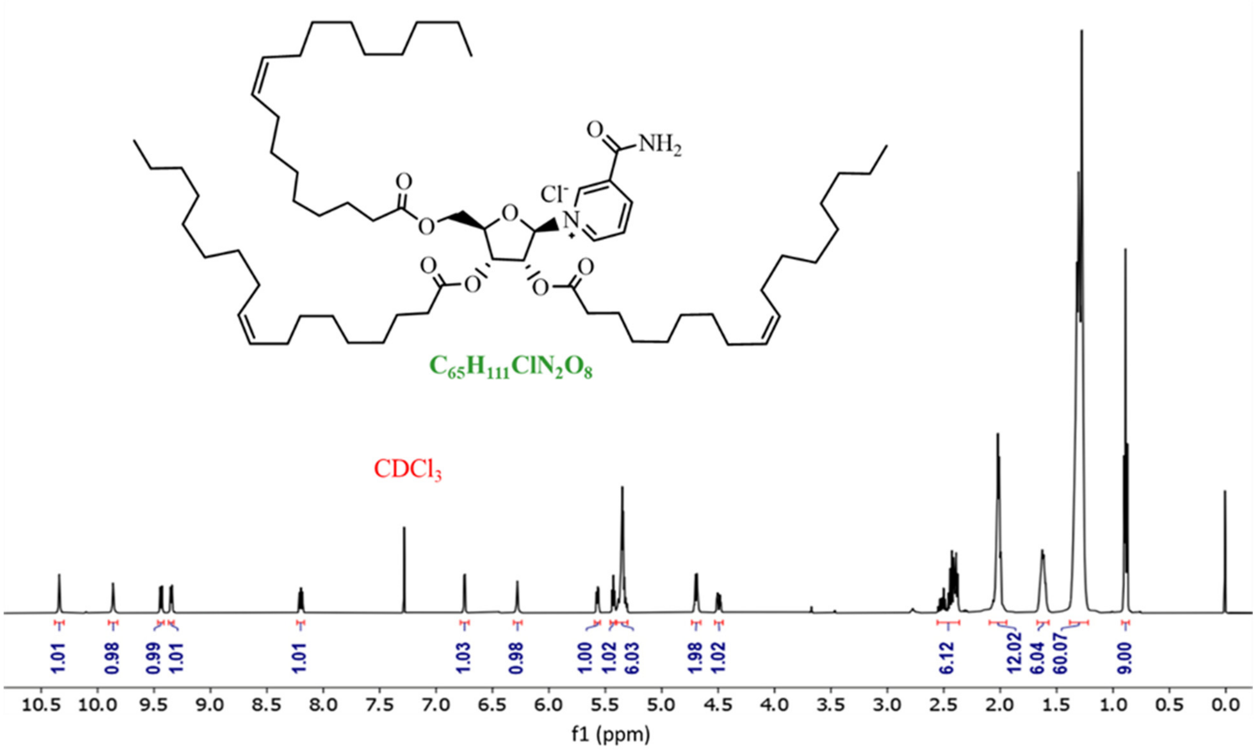

Figure S2. ^{1}H NMR of NRTOCl in $CDCl_3$.

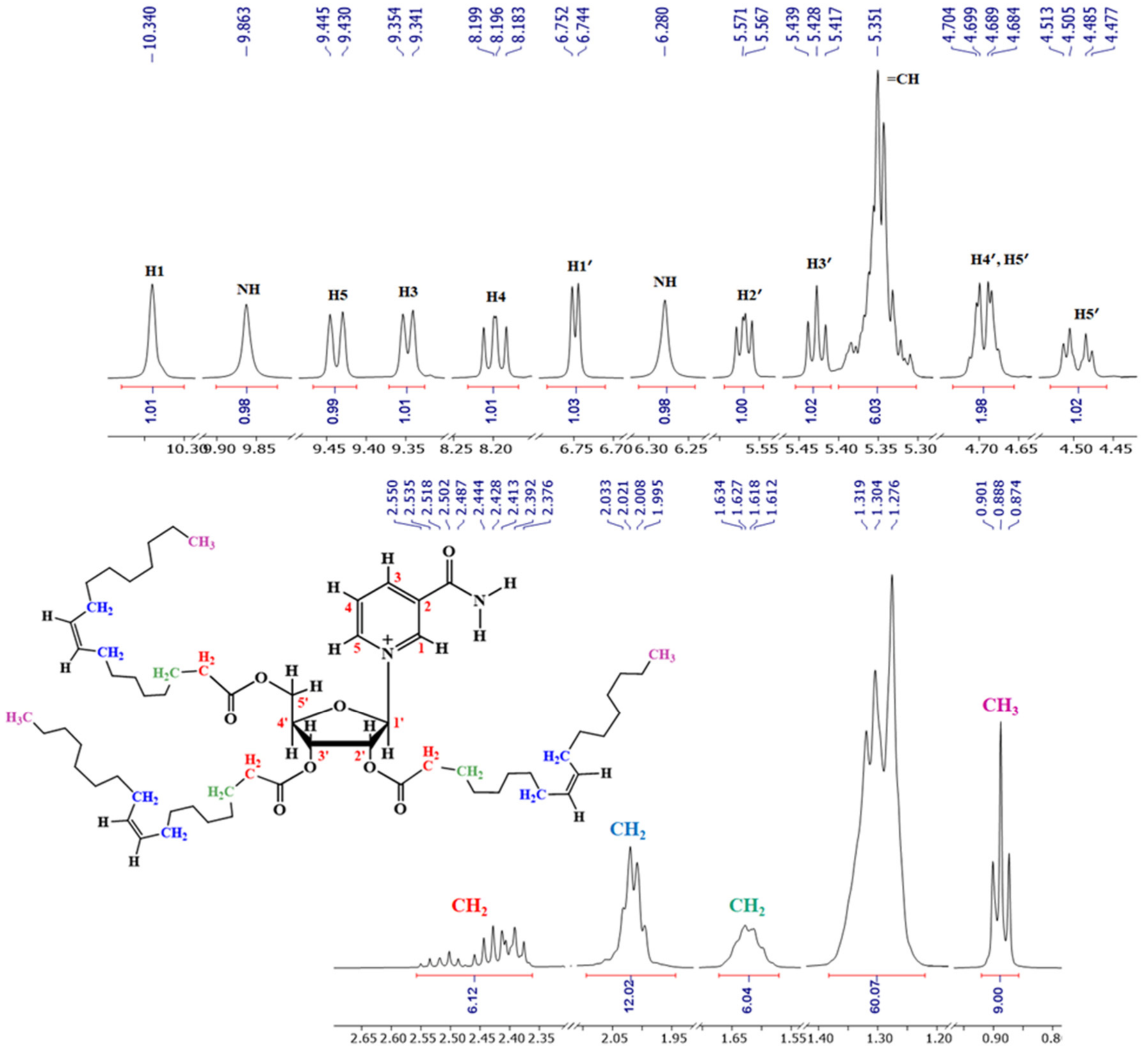

Figure S3. Expanded ^{1}H NMR of NRTOCl.

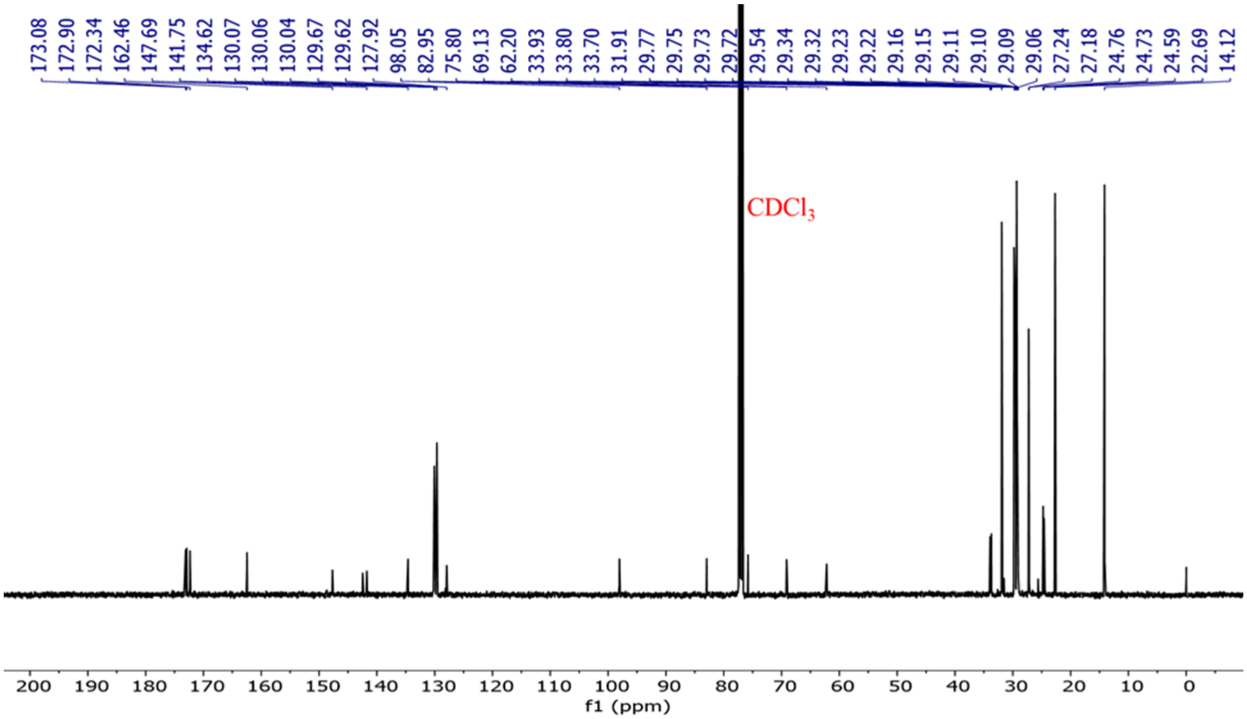

Figure S4. ^{13}C NMR of NRTOCl in $CDCl_3$.

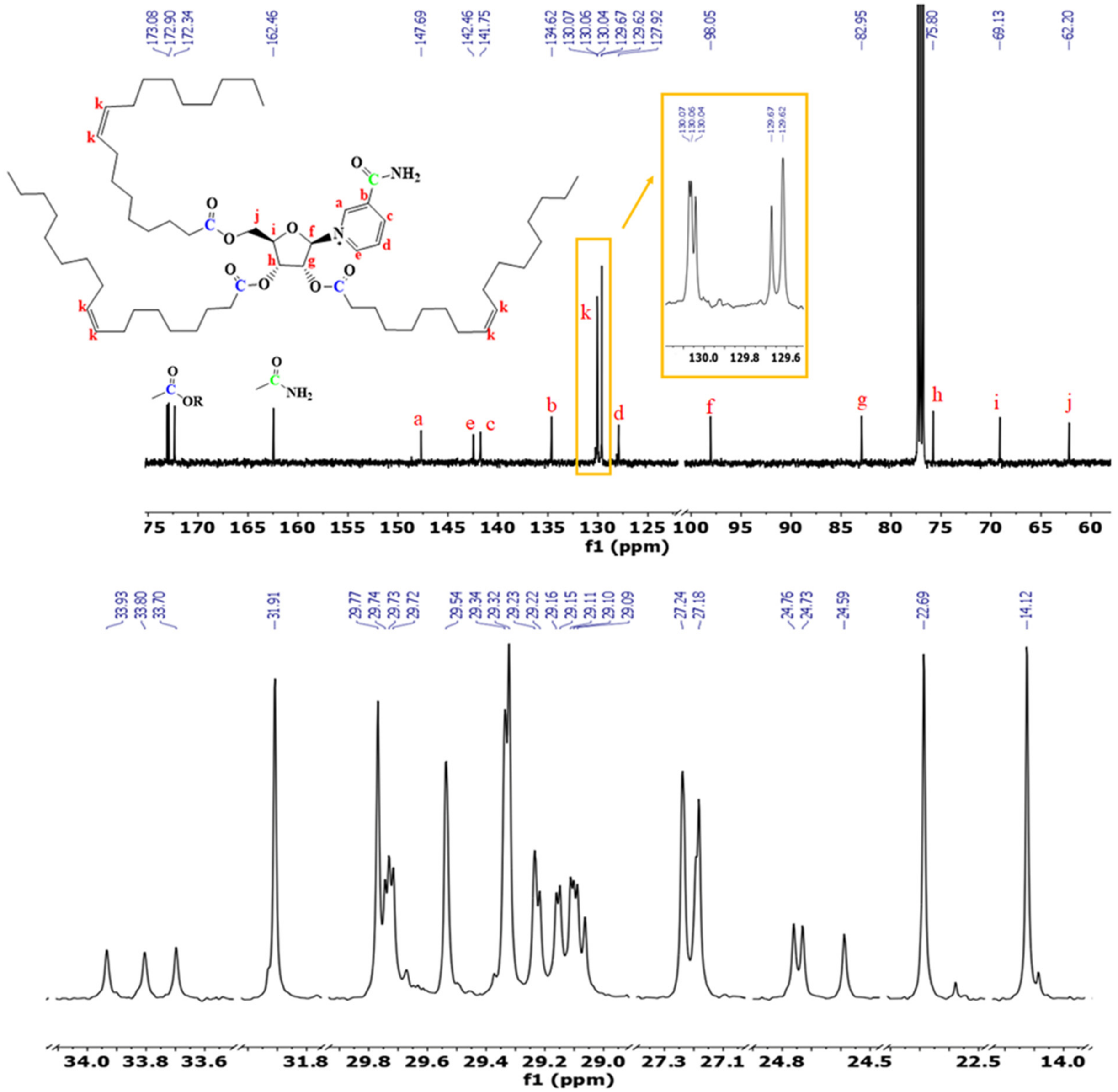

Figure S5. Expanded ^{13}C NMR of NRTOCl.

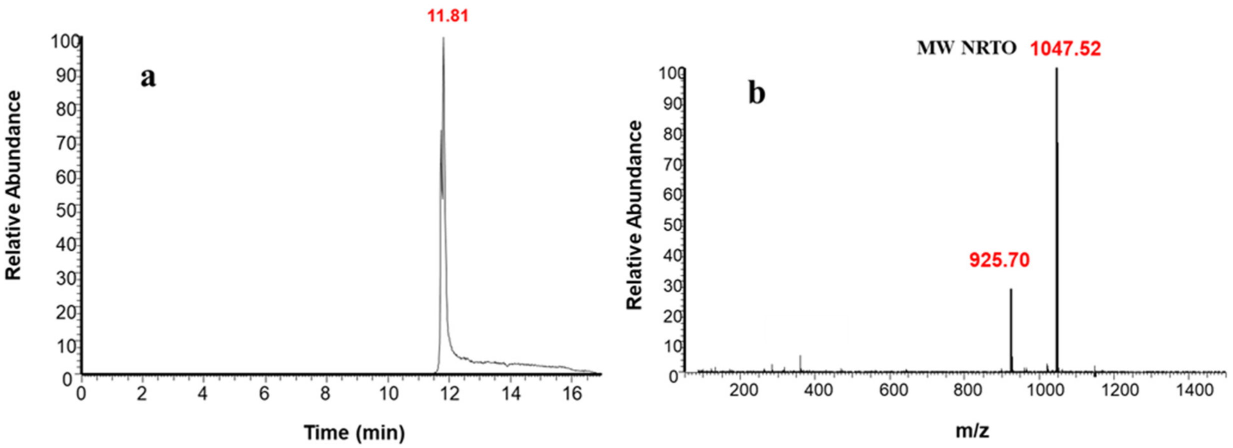

Figure S6. SRM LC-MS of NRTOCl. a) SRM LC of NRTOCl. b) Mass spectrum of NRTOCl.

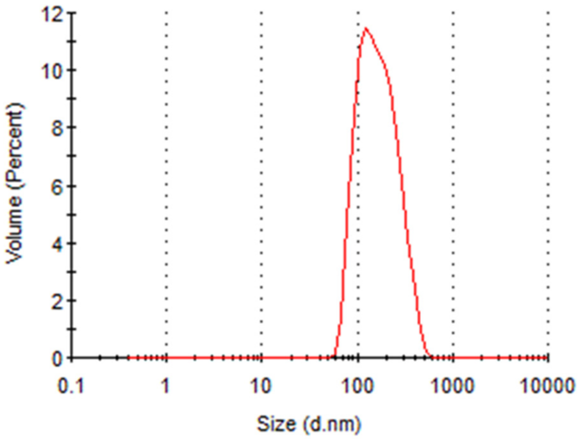

Figure S7. Particle size of NRTOCl in DI water containing 1 % (v/v) ethanol.
